# Advanced Technologies for Characterizing and Detecting Battery Thermal Failure: A Review

**DOI:** 10.1002/advs.202600050

**Published:** 2026-05-08

**Authors:** Yongxiu Chen, Zeyu Sun, Wei Zong, Yuhang Dai, Kai Ling Ng, Paul R. Shearing

**Affiliations:** ^1^ Department of Engineering Science University of Oxford Oxford UK; ^2^ The Faraday Institution Quad One Becquerel Avenue Harwell Campus Didcot UK; ^3^ The ZERO Institute Holywell House Osney Mead Oxford UK

**Keywords:** acoustic imaging, lithium‐ion batteries, optical sensing, thermal failure, X‐ray imaging

## Abstract

Energy storage is essential in accelerating the global transition toward clean and sustainable energy across various sectors. Lithium‐ion batteries (LIBs) have become increasingly significant for energy storage due to their high energy density, low maintenance, and high coulombic efficiency. However, a challenge arises from the risk of thermal failure across all cell formats and chemistries, particularly under harsh electrochemical and thermal conditions and mechanical stress. In severe cases, such failures have led to catastrophic fires or explosions, posing substantial safety risks and environmental concerns due to gas emissions and toxic discharge. Therefore, an understanding of thermal failure mechanisms is essential for ensuring the safe deployment of batteries across various applications. This review first examines the causes and consequences of thermal failure, providing didactic mechanistic insights. It then compares recent progress in thermal and electrochemical failure analysis using advanced characterization techniques, including X‐ray imaging, optical sensing, acoustic field imaging, and spectroscopy, to study the thermal runaway (TR) mechanism and reveal how thermal failures develop and propagate in batteries. It further examines how these advanced characterization tools can inform battery management systems and industrial practice and concludes by outlining future challenges and perspectives for achieving safe energy storage systems.

## Graphical abstract

Illustration of advanced characterization techniques to predict battery thermal failure.

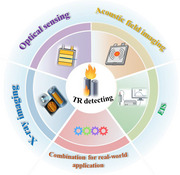

## 1 Introduction

The Paris Agreement, adopted by 196 parties in 2015, has set the goal of achieving net‐zero between 2055 and 2080 [1, 2, 3, 4]. In this regard, the UK has been one of the leading players in the transition to green energy with legally binding commitments to decarbonize [5]. To support the energy transition, batteries (including LIBs and SIBs) have played a leading role in energy storage for electrified transport and low‐carbon grids [6, 7, 8, 9]. Benefiting from their high energy/power density, these rechargeable batteries are widely used to power electrified transport (ranging from micro‐mobility to EVs, locomotives, and ships) and large‐scale energy storage (national and local grids, etc.), as well as for ubiquitous applications in consumer electronics [10, 11, 12, 13]. Whilst ‘in‐field’ failures are rare, under extreme conditions (e.g., high temperature, penetration, crushing, or overcharging), battery energy storage can lead to serious fires in workplaces, residential buildings, and highways globally [14, 15, 16]. Thermal runaway (TR) refers to an uncontrolled, self‐accelerating reaction within a battery that can lead to overheating, fire, or explosion, which is typically triggered by external heat, mechanical damage, or electrical abuse. Therefore, this real‐world evidence underscores the need for improved understanding of TR failures and strategies to mitigate them [14, 17].

Numerous innovations have been made to prevent, delay, or suppress the above TR in rechargeable batteries at varying design levels. First, thermo‐responsive binders and separators have been deployed and act through phase‑change polymers that shut down ion flux to prevent TR initiation [18, 19, 20]. Similarly, a safety‐reinforced layer (SRL) on current collectors has been introduced to prevent current overflow, thus preventing further exothermic reactions in the event of battery failure [21]. Electrolyte modifications (i.e., non‑flammable, fluorinated, or high‑concentration ‘localized’ electrolytes) have been proposed to suppress gas evolution in liquid electrolytes, shift the onset temperature of exothermic reactions, and stabilize the cathode from oxygen loss [22, 23, 24, 25]. Solid‐state batteries, which remove flammable liquid electrolytes, also significantly mitigate thermal hazards [26]. Following this, the cell‐level innovations include safety‐vents via valve architectures that allow gas venting once the battery internal pressure reaches the threshold, current interrupt devices (CID), and increasingly sophisticated systems for early TR warning of the battery [27, 28, 29]. Battery thermal‑management systems (BTMS) have also adopted phase‐change material (PCM) integrated between cells to absorb heat generated during battery abuse and failure [30]. The examples above illustrate some of the multi‐scale innovations designed to suppress thermal failure. To inform the overall understanding and guide future research directions for TR mitigation, numerous recent reviews explore mitigations to solve battery TR [31, 32], the strategy for monitoring physical and electrochemical parameters [33], and the approaches for battery TR modelling and diagnosis [34, 35].

Additionally, early detection of battery issues through physical (stress, swelling, cracks, etc.), chemical (gas emission), thermal, and electrochemical (voltage, resistance, etc.) signals has been used for real‐time, non‐invasive identification of thermal failure precursors via advanced characterization technologies (such as X‐ray, acoustic sensing). These methods offer powerful tools for monitoring internal battery structure changes, degradation mechanisms (e.g., lithium plating, SEI breakdown, dendrite formation, and gas evolution), and thermal progression. However, the current review literature lacks a comprehensive overview of real‐time detection of TR through the deployment of these characterization technologies. Further understanding of this area of battery science is crucial for comprehending the relationship between battery degradation and thermal failure.

Motivated by the above gaps, in this review, we first examine the causes of thermal failure, providing insights into its propagation mechanisms. Then, we compare advanced methodologies for triggering and observing TR, illustrating how thermal failures develop and progress in batteries. Furthermore, we discuss technologies designed to detect and predict thermal events before TR occurs. Finally, the review highlights future challenges and offers perspectives to guide ongoing efforts toward the safe application of battery technologies.

## 2 Thermal Runaway

**FIGURE 1:**
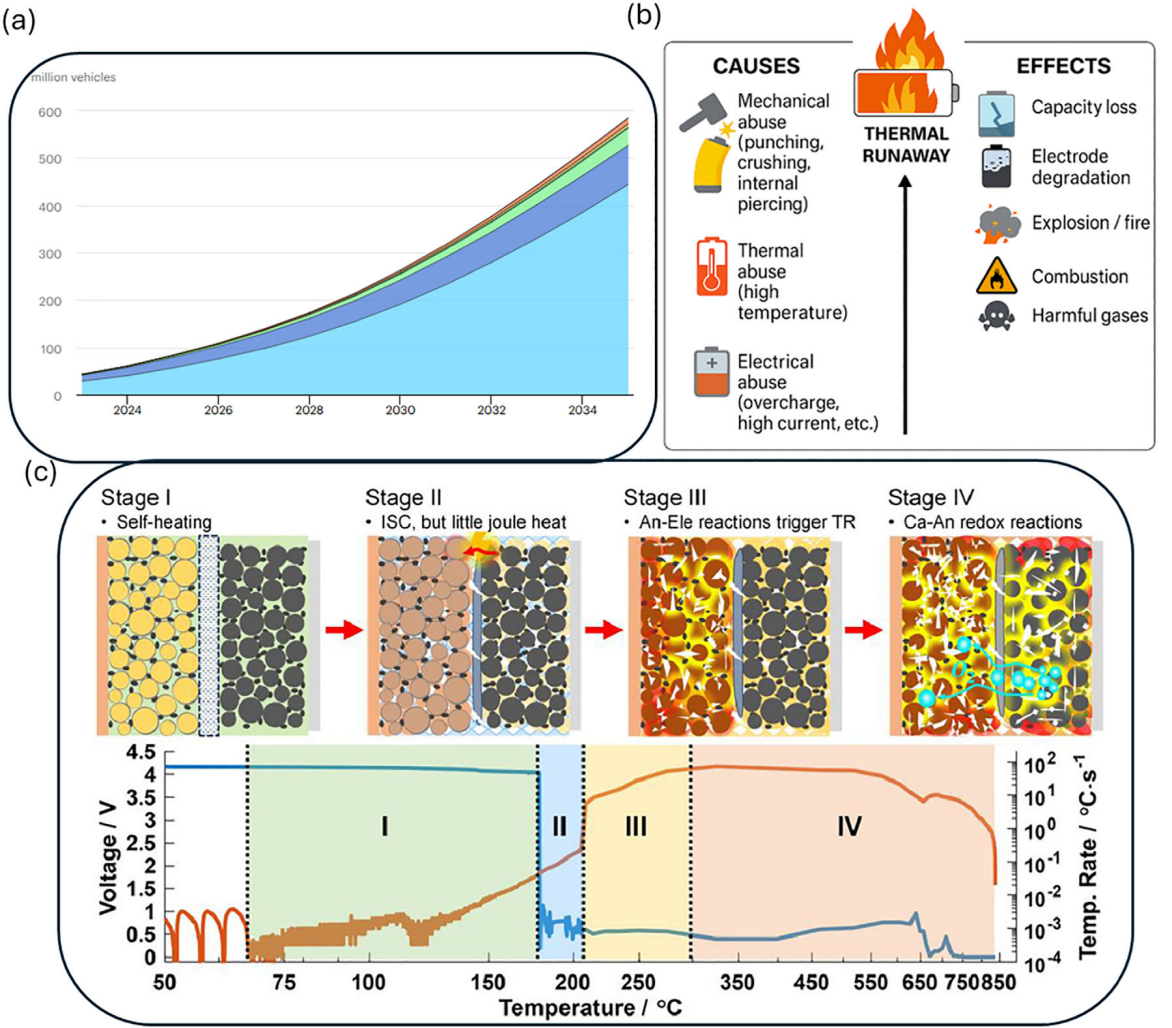
(a) Projection of global EV stock. Reproduced from [36], licensed under CC BY 4.0. Copyright 2024 International Energy Agency (IEA). (b) Initiating causes and hazards of battery thermal runaway. (c) Mechanism and evolution of TR in the battery. Reproduced with permission [37]. Copyright 2021, Elsevier.

We have witnessed an inexorable rise in real‐world applications of batteries. The global population of EVs across all modes is expected to grow from less than 45 million in 2023 to 250 million in 2030, and reach 525 million in 2035, as illustrated in Figure 1a [36]. However, when these batteries suffer from an internal short circuit or external thermal abuse, a sequence of exothermic events may be triggered. When the heat generated inside a cell exceeds the heat dissipated to the environment, catastrophic thermal failure can occur. Once external stimuli (e.g., mechanical abuse, electrical abuse, thermal abuse) trigger a thermal event, it not only accelerates battery thermal degradation (e.g., electrolyte decomposition, SEI breakdown, and electrode reactions) but may also result in flammable gas venting, fires, or even explosions. Whilst the risk of thermal failure is rare, its occurrence has been documented across a range of applications, hindering further growth in battery deployment across sectors and may compromise public safety (Figure 1b).

### 2.1 Thermal Runaway Evolution in a Single Cell

In theory, the progression of TR involves several sequential stages, as shown in Figure 1c [37, 38]. Stage I: The TR begins during the onset phase of self‐heating triggered by an event (such as overcharging, overheating, or mechanical abuse). This localized or whole‐cell heating initiates uncontrolled, self‐sustaining reactions in the battery. In this stage, the thermal breakdown of the solid electrolyte interphase (SEI) typically occurs at 50 °C–70 °C, thus contributing to the heat and gases released.

Stage II: the above local heating may lead to the shrinking of separators, with continually generated Joule heating reaching ∼200 °C.

Stage III: Exothermic reactions between the anode and the electrolyte proceed and accelerate at elevated temperatures, generating considerable heat. Gas formation contributes to continual internal pressure buildup.

Stage IV: The active metal ions (e.g., Co^4+^, Ni^4+^) are dissolved into the electrode pores and reduced, or the oxygen is released from the active material crystal lattice. These initiated redox reactions at the electrode are uncontrolled or spontaneous and involve extreme heat generation, rapidly bringing the battery to an extremely high temperature.

Stage V: Finally, the TR process can lead to cell fire, venting, or explosion.

### 2.2 Thermal Runaway Propagation in the Pack‐Level Cells

Once TR is initiated in a single cell, it can spread to neighboring cells, ultimately leading to battery‐pack‐scale thermal failure. Its propagation results from the complex coupling among thermal, electrical, mechanical, and chemical processes.

First, the most direct route for propagation is heat transfer from the failed cell to adjacent cells. In this scenario, the neighboring cells would absorb a large amount of heat through metal interconnects or structural interfaces within the battery module [39]. In this step, the cell geometry, contact resistance, and thermal conductivity of the battery module casing will strongly influence propagation dynamics. When the neighboring cell surface temperature exceeds critical thresholds (typically, 130 °C–150 °C), its internal components begin to degrade, and TR is initiated in that cell. This sharpens the focus on the importance of external heating as a trigger for TR and its propagation from an initial cell to its neighbors. Advanced X‐ray imaging has revealed that structural designs (e.g., poor ventilation or high packing density) may exacerbate heat transfer and accelerate the propagation of thermal failure [28]. To avoid thermal propagation by heat transfer between cells, increasing cell spacing or using thermal insulation materials (radiant barrier and intumescent materials) can effectively protect surrounding cells from heat released during TR.

Following this, flammable gases such as hydrogen, methane, carbon monoxide, and hydrocarbons might be released, which can ignite and cause thermal failure in neighboring cells. If the battery casing is compromised during TR, vented gases may quickly build up and could ignite if there are nearby ignition sources or high temperatures. Additionally, the intense heat generated during jet flame or explosive events can damage the separator of nearby cells and might ignite already‐leaked electrolytes, leading to further fire spread throughout the entire battery pack.

A local short circuit is another cause of battery thermal failure and its spread within the battery pack or module. These may result from electrical abuse (such as high current leading to lithium dendrite formation), mechanical abuse (like collisions and compression during EV accidents), or design flaws (such as separator breakage during thermal battery assembly). This can cause localized high current flow and rapid heat buildup in the battery. In this context, thermal expansion or mechanical rupture during TR can pierce nearby cells, leading to internal short circuits and triggering TR again. Additionally, Liu et al. compared propagation risks across different short‐circuit initiation methods (including triggering with phase‐change materials, shape‐memory alloys, artificially induced dendrite growth, equivalent resistance, and nail penetration), and found that mechanically induced short circuits are particularly common causes of thermal failure spread [40].

Several critical battery design factors are also at high risk of accelerating the propagation of battery thermal failure. These include high cell density, lack of insulation between cells, ineffective heat‐dissipation paths (i.e., poor thermal interfaces or blocked vents), and a poor BMS that fails to detect abnormal conditions sufficiently quickly [31, 38]. When heat dissipation is enhanced to prevent rapid temperature buildup in the cell, the possibility of TR propagation is reduced.

## 3 Experimental Lab‐Scale Observing and Quantifying TR

To understand how the TR evolves and assess cell safety, many methods have been employed to trigger and measure battery thermal failure, aiming to predict the thermal behaviors of batteries in real‐world applications. Generally, these abuse tests (mechanical, electrical, thermal, or manufacturing defects) would cause battery internal short circuit (ISC), resulting in rapid increases both in temperature and pressure. Observing and quantifying these behaviors can guide future research on material innovations and help advance battery development toward safer designs.

### 3.1 Mechanical Design

The safety of LIBs under mechanical abuse remains a critical concern in real‐world scenarios. Mechanical events, such as collisions, indentation, and compression, can trigger a series of internal failure mechanisms that lead to TR. Nail penetration tests, widely deployed in cell certification, combine elements of mechanical and electrical abuse, whilst crush and indentation testing principally replicate the effects of mechanical events (which may in turn lead to ISC).

#### 3.1.1 Collision and Compression

During mechanical abuse, the external forces exerted on the battery can cause electrode bending, separator rupture, or internal short circuits. Normally, regulatory safety evaluations involve simulating mechanical abuse scenarios such as vehicular collisions and accidental drops. The corresponding testing protocols can lead to the rapid deformation of the battery structural elements, resulting in complex interactions between macroscopic collapse and microscopic failure mechanisms. Therefore, these procedures can serve as a fundamental basis for evaluating the mechanical resilience and overall safety performance of battery systems.

Compression and impact tests are intended to simulate large‐scale structural failures under more severe external mechanical loading. Standardized tests are used to replicate mechanical events such as vehicular collisions and accidental drops, which can lead to rapid structural collapse and complex internal damage, ultimately initiating TR. These scenarios result in overall collapse or distortion of the battery mechanical housing and internal layers, potentially causing internal electrical contact due to separator damage and layer displacement. Such failures are typically accompanied by rapid heat generation, violent emission of flammable gases, and substantial material ejection [41, 42].

Unlike compression or impact tests, indentation testing is used to evaluate localized mechanical failure by applying a concentrated compressive force over a confined area, thereby simulating the effects of point loading, localized mechanical stress, and structural deformation of the battery under external compressive stress [43]. Such deformation within the multilayer architecture may induce minor ISCs or localized contact failure. Maleki et al. investigated the influence of ISC location on the thermal behavior of prismatic LIBs using surface indentation and pinching techniques (in addition to small‐nail penetration) [44]. They demonstrated that high‐risk ISC events are more likely to occur near electrode edges and internal structural interfaces, particularly under pinching and indentation conditions. This highlights further improvements in the mechanical stability of the electrode edges in the battery design.

#### 3.1.2 Nail Penetration

Although the nail penetration test has limited relevance to expected real‐world failures, it provides a facile means of driving simultaneous mechanical abuse and ISC. To systematically evaluate and mitigate such risks, international regulatory standards (including IEC TR 62660‐4 and GB38031) have been established, providing guidelines for consistent assessment of battery resilience to ISC events [45, 46, 47, 48].

**FIGURE 2:**
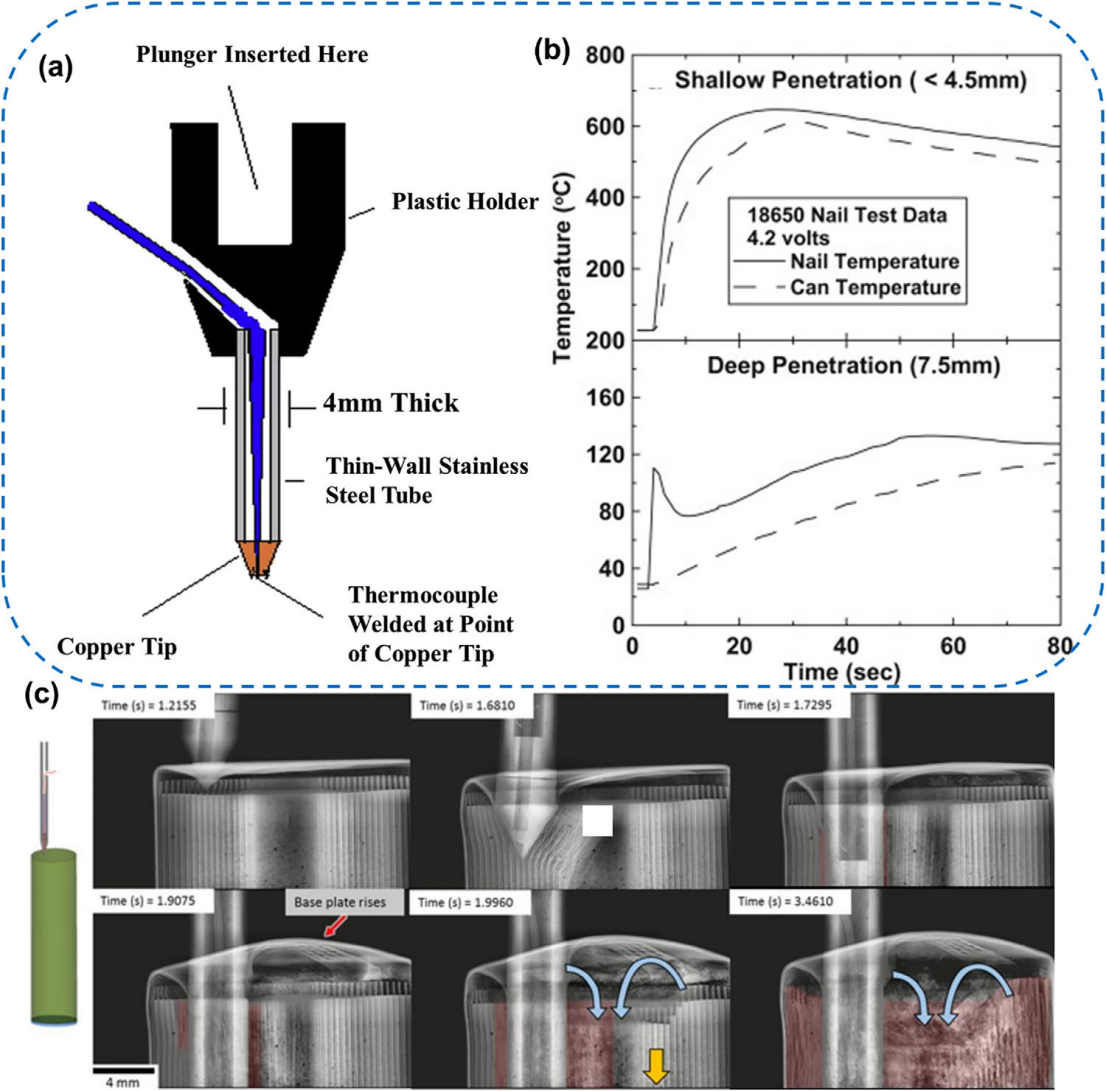
Cases of penetration trigger to observe the battery TR: internal smart nail design (a,b) to trigger battery TR (Reproduced with permission [49], Copyright 2013, Elsevier.) and time‐stamped radiographs. (c) nail penetration showing initiation and propagation of TR. Reproduced with permission under the terms Creative Commons Attribution 4.0 License [77]. Copyright 2017, The author(s), Published by ECS.

To improve understanding of thermal behavior during ISC in LIBs, Dahn et al. developed a novel diagnostic design known as the ‘smart nail’ [49]. This device consists of a hollow stainless‐steel shaft with a sharpened copper tip, into which a thermocouple is embedded to measure internal cell temperatures precisely at the point of penetration (Figure 2a). The design enables real‐time monitoring of the thermal response during mechanical abuse testing by recording the temperature evolution inside the cell, in contrast to traditional external sensors (Figure 2b). These internal measurements offer critical insights into the initiation and development of TR, thereby improving the accuracy of failure models and safety assessments. In addition to electrical connections to the cell terminals, the smart nail also enables simultaneous voltage monitoring, enabling a comprehensive evaluation of both thermal and electrical responses during failure events. Despite offering internal state monitoring of the LIB, the smart nail design has several limitations. The metallic shaft can dissipate heat from the reaction zone, potentially underestimating the peak temperature. In addition, the temperature measurement is confined to a single point along the penetration path, thus limiting spatial resolution. Additionally, variations in penetration depth and alignment can affect sensor contact and reduce data accuracy and consistency. Nevertheless, the smart nail remains a valuable diagnostic tool for battery safety research. If combined with complementary sensing techniques, it contributes to a more comprehensive and spatially resolved thermal profile under abusive conditions.

Building on the smart nail concept described above, Finegan et al. integrated thermocouples into penetration needles and combined this setup with high‐speed X‐ray imaging to accurately monitor the evolution of internal temperature and structural degradation during mechanical abuse. This study confirmed that ISC can quickly trigger TR, characterized by intense heat generation, gas release, and internal shock currents that weaken the cell structural integrity. Moreover, it was found that the jelly‐roll structure of cylindrical cells gradually increases the effective conduction area during axial nail penetration. This structural feature partly reduces the severity of ISC, leading to a slower voltage drop compared to axial penetration, as shown in Figure 2c [50].

However, the results of battery penetration testing have poor repeatability. To improve this, the penetration speed, penetrator material, and battery state of charge have been investigated. First, Diekmann et al. [51] highlighted the impact of penetration speed on TR outcomes and found that slower penetration rates (approximately 1 mm s^−1^) improved test repeatability and TR initiation stability in pouch cells. Huang et al. further developed a small, slow, and in situ sensing nail penetration method, enabling detailed observation of ISC initiation with good repeatability and subsequent TR processes [52]. Second, the penetrator material also affects the TR behavior in the battery. The metallic penetrators were found to accelerate TR at higher SOC levels due to rapid Joule heating and their conductive nature. In contrast, ceramic penetrators, with low thermal conductivity and electrical insulation properties, were less likely to trigger TR at lower SOC levels. This methodology provides valuable insights for designing a tool to trigger TR. However, the outcomes vary widely depending on the position, speed, and direction of nail indentation. If a nail enters through the positive terminal of a cylindrical cell, it can block a vent path. Therefore, accurately piecing the same position on the cells is fundamental to achieving high data consistency [53, 54]. Third, the TR behavior of LIBs in nail penetration testing exhibits strong dependence on SOC [69]. Yang et al. conducted penetration tests on eight types of commercial LFP cells and found that, as SOC increases, both mass loss and peak temperature during TR increase accordingly [42, 55]. Chen et al. employed radial nail penetration to trigger TR, demonstrating that the severity of sidewall rupture strongly depends on the SOC. At SOC ≥ 50 %, rapid gas release caused a significant rise in internal pressure and increased the risk of rupture [56]. The subsequent ejection of flammable gases can further induce deflagration and jet flame phenomena. In contrast, SOC < 50 % corresponds to a lower probability and reduced extent of rupture.

In summary, research on mechanical abuse‐induced TR in LIB has substantially advanced the understanding of failure mechanisms and influencing factors. Existing studies have elucidated the multifaceted effects of mechanical events on internal short‐circuit formation, gas evolution, and the progression of TR, thereby facilitating the development of relevant diagnostic and testing methodologies. However, the fundamental principles governing multi‐physics coupling under varying mechanical loading conditions remain insufficiently understood, and standardized, reproducible testing systems and associated models/digital twins are yet to be established. Future efforts should integrate advanced in situ characterization techniques with multi‐scale modelling to further elucidate the intrinsic relationship between mechanical damage and TR.

### 3.2 Electrical Design

Overcharge, over‐discharge, and external short circuit (ESC) in LIBs are three triggers of battery TR. Here, we summarize current methodologies and evaluate their effectiveness and limitations.

#### 3.2.1 Overcharge

**FIGURE 3:**
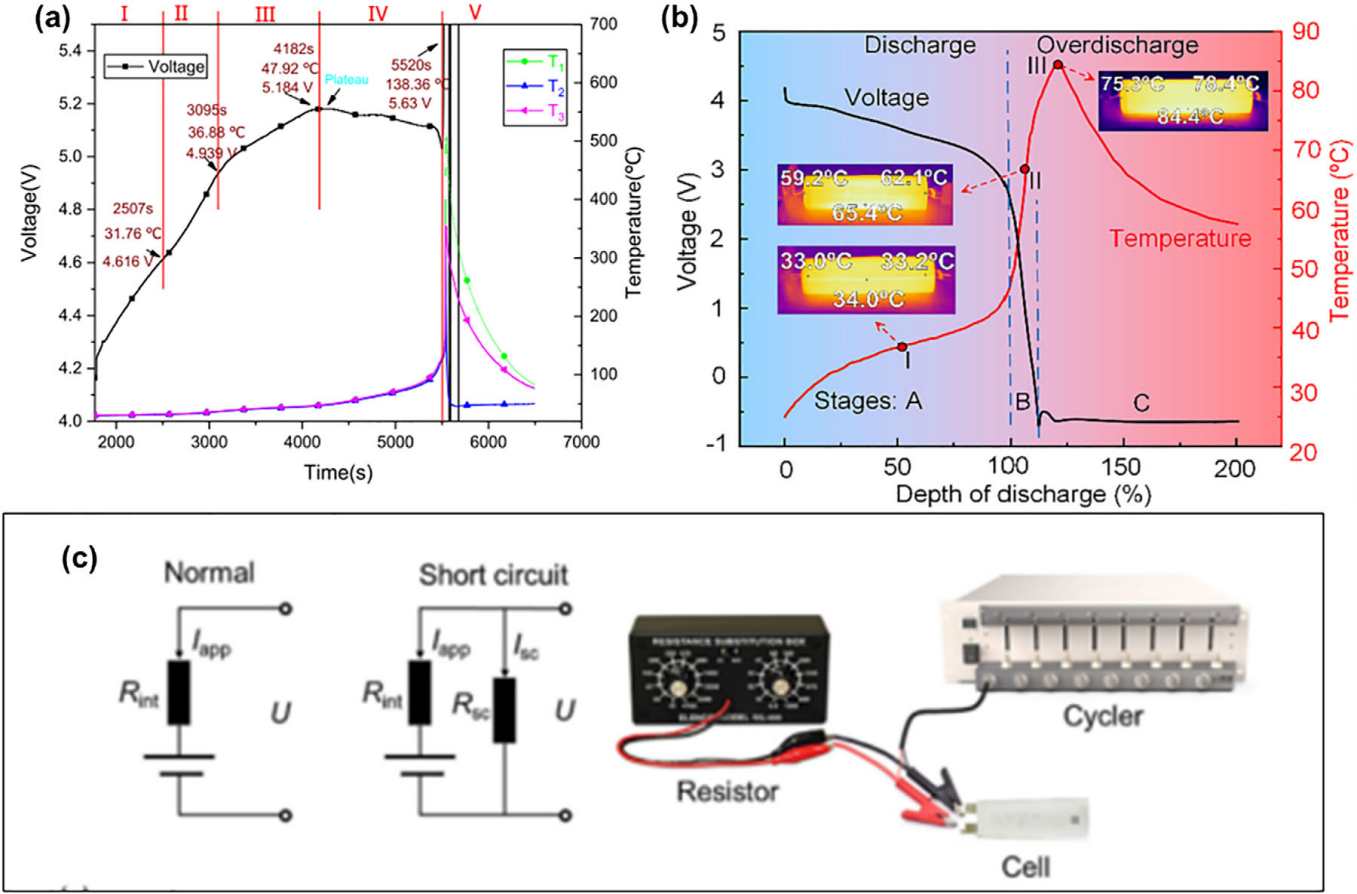
Electrical design to observe and quantify the battery TR via : (a) overcharge. Reproduced with permission [60]. Copyright 2022, Elsevier. (b) over discharge, Reproduced with permission [66]. Copyright 2023, Elsevier. (c) external short circuits, Reproduced with permission [72]. Copyright 2023, Elsevier.

Overcharge is a form of electrical abuse that triggers abnormal electrochemical processes and drives internal imbalance in the battery, as shown in Figure 3a. This can lead to the growth of lithium dendrites, which induce ISC and interfacial degradation, ultimately resulting in battery failure. Lithium dendrite formation typically occurs during battery (over)charging, primarily due to non‐uniform current distribution and surface irregularities on the anode, which hinder the uniform intercalation of lithium ions into the graphite structure. This is exacerbated at low temperatures and in cases of local cell imbalance, where surplus lithium ions cannot intercalate into the graphite lattice and consequently undergo electrochemical reduction to form the metallic lithium on the anode surface. This metallic lithium progressively forms dendritic structures, which can puncture the separators and cause ISC. These ISC events are influenced significantly by electrode and electrolyte properties, which may produce localized regions of elevated current densities, form thermal hotspots, and trigger battery thermal failure. These ISCs can be further classified into hard ISCs, which are irreversible due to a continuous voltage drop, and soft ISCs, which are reversible because the voltage can recover to its normal level [57]. However, such soft shorts (resulting, e.g., from Li dendrite formation) can ‘mature’ into hard shorts with repeated plating. To prevent both hard and soft shorts from dendrite nucleation and evolution during overcharging, a deliberate cell balance (i.e., a high N:P ratio) provides a buffer, enabling uniform plating, robust interphases, mechanical resilience, and the prevention of dead‐Li formation due to the increasing active surface area and lithiation sites. This suppresses the probability of dendrite growth and significantly reduces the risk of TR.

Apart from the undesired formation of lithium dendrites, overcharging also induces a series of physicochemical instabilities at the interface, further compromising battery performance and safety. In investigations of high‐capacity NMC batteries, the elevated electrochemical potential induced by overcharging significantly accelerates the dissolution of manganese ions (Mn^2+^) from composite cathode materials [58]. These metal ions would interfere with the solid electrolyte interphase (SEI) at the anode, causing structural instability and accelerating parasitic reactions that degrade battery capacity [59]. The elevated voltage promotes oxidative decomposition of the electrolyte, with Ohmic heating and exothermic side reactions further aggravating heat and gas generation [59, 60]. Moreover, over‐delithiation of cathodes, particularly those with high nickel content, can induce structural collapse and oxygen release. The released oxygen exacerbates electrolyte decomposition and generates significant amounts of gas, increasing cell resistance and internal temperatures, ultimately raising cell pressure and further cell rupture [61]. Furthermore, upon venting, the released gases may pose public health and environmental concerns [62].

To enhance the thermal safety of LIBs under overcharge conditions, commercial battery systems commonly adopt possible protective methodologies, such as positive temperature coefficient (PTC) thermistors and current interrupt devices (CIDs), particularly in cylindrical cell configurations [63, 64]. The PTCs would work by increasing their internal resistance as temperature rises, thereby limiting current flow and suppressing excessive heat generation. In addition, the CIDs operate by mechanically breaking the internal circuit once the internal pressure exceeds a defined safety threshold. Therefore, integrating these components mitigates the consequences of TR by limiting further thermal escalation and reducing the likelihood of catastrophic failure.

#### 3.2.2 Over‐Discharge

As another form of electrical abuse, over‐discharge can lead to spatial heterogeneity in electrochemical performance. In this case, any cells in a pack that fall below their specified cutoff voltage would damage the whole pack cycling performance even before the entire pack reaches its lower limit. In the absence of BMS monitoring or intervention, these cells keep discharging, and electrical abuse can occur [62, 65]. This abuse triggers thermodynamically unstable reduction reactions, resulting in the electrochemical dissolution of copper at lower SoC (voltage <−1.2 V with the range of −12 %–0 %) from the anode current collector in Figure 3b [66]. The dissolved copper (Cu^2^^+^) originating from the dissolution of the anode current collector has been found to migrate across the separator and deposit on the cathode in the form of metallic dendrites(voltage < −1.77 V with SoC range of −13 %–−15 %), which compromises both cathode and anode cycling performance and causes a possible internal circuit due to the metallic copper dendrites [67]. This progressive dendrite growth may also breach the separator and cause internal short circuits with localized heat generation [68].

To fully understand the overcharge/discharge, both the cell capacity and electrode thickness are found to affect battery thermal behavior. In this regard, high‐capacity LIBs exhibit much greater sensitivity to both overcharging and over‐discharging [66, 69]. This is primarily attributed to their larger volume and extended thermal diffusion paths, which exacerbate localized heat accumulation and temperature gradients during electrical abuse. Then, the thicker electrode structures in high‐capacity cells increase ionic transport resistance and current density heterogeneity, promoting spatially uneven electrochemical reactions and localized lithium plating, thereby compromising overall cell performance and safety. To avoid the above over‐discharge, the main interventions should seek to protect the anode from dendrites and dissolved Cu current collectors. Possible solutions could include accurate cell‐level voltage or SoC monitoring/estimation, moderate N:P cell balancing (N:P 1.1–1.3), and conservative discharge constant protocols that are higher than the lower limit of the discharge voltage (i.e., SoC ≥ 10 %).

#### 3.2.3 External Short Circuits

External short circuits (ESCs) pose serious safety risks and are included in international certification protocols as standard tests (i.e., IEC 62619:2022) [70]. They are typically caused by defects in electrical connectors or by mechanical failures that create unintended conductive pathways (see Figure 3c). When an unintended external conductive pathway forms, bypassing the designated circuit can result in an abrupt, substantial current surge that induces severe internal heating within the cell. An et al. simulated ESC by applying an ultra‐high discharge current at a rate of 20 C [71]. It was observed that cells with thicker electrodes exhibited particle fracture, separator shrinkage, and increased surface deposition on the negative electrode following the ESC event. Jia et al. also systematically conducted ESC tests to generate controlled fault conditions and construct a large‐scale dataset, enabling the development of a data‐driven model for estimating ESC resistance [72].

In addition, unstable electrical connections can induce series arc faults, which generate high‐energy arcs and intense localized heating. Such faults are more likely to occur in high‐voltage battery packs and are exacerbated by conductive gas venting during failure. The intense local heating from arc faults significantly compromises internal battery integrity, especially when flammable electrolytes are exposed to external oxygen. Zhang et al. [73] simulated arc faults by striking a tungsten needle against the safety valve of a 40 Ah LiFePO_4_ prismatic cell using a custom‐built arc initiation apparatus. The setup consisted of a 400V/20 A DC regulated power supply connected through a high‐power resistor and controlled via a vertical sliding mechanism to initiate the arc. The results indicate that the extent of structural damage is primarily determined by the instantaneous arc power rather than the total arc energy. This suggests that even short‐duration arcs can generate extremely high local temperatures, leading to the melting of critical components and the loss of seal integrity.

### 3.3 Thermal Abuse

When a battery cell is exposed to external heat, the supplied thermal energy can activate a series of exothermic reactions. These reactions include the thermal decomposition of battery components and subsequent reactions between the decomposed materials and the electrolyte. Once initiated, the heat released from these reactions further accelerates additional exothermic processes, creating a self‐reinforcing cycle of heat generation. This negative feedback loop continues, leading to TR. Consequently, battery failures that breach the onset of TR are self‐sustained and persist until all reactive materials are depleted. Thermal abuse is a key mechanism for TR propagation in modules and packs, wherein the first ‘trigger’ cell to fail within a module can generate sufficient heat to provoke thermally abusive conditions in neighboring cells.

**FIGURE 4:**
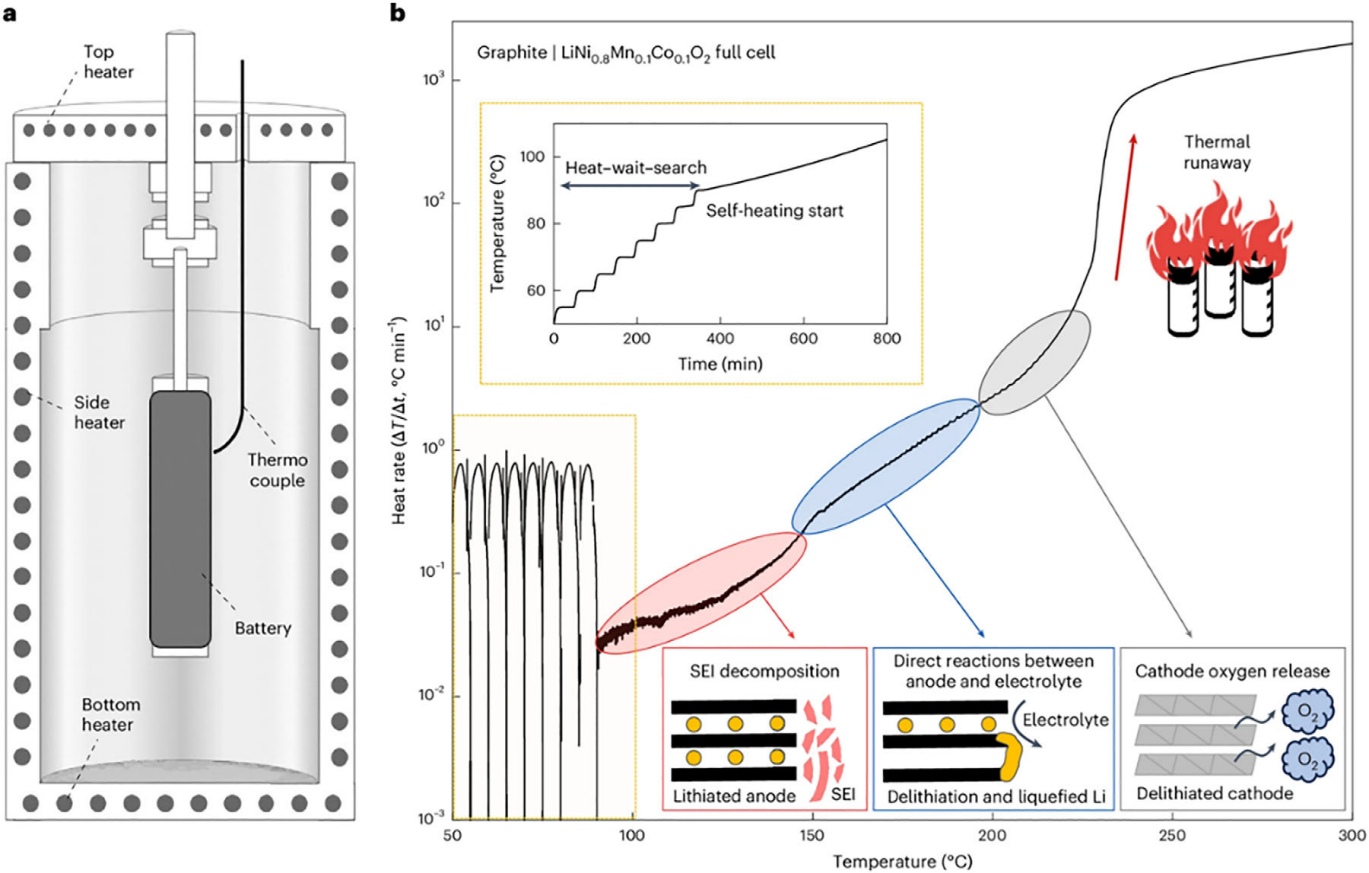
ARC tests to investigate NMC//Gr cellTR and battery degradation mechanism. Reproduced with permission under the terms Creative Commons CC‐BY‐NC‐ND license [74]. Copyright 2025, The author(s), Published by Spring Nature.

The accelerating rate calorimeter (ARC) has been widely used as a thermal abuse test to heat the cell under adiabatic conditions, thereby triggering the evolution of battery thermal failure, as shown in Figure 4. At the initial stage, the SEI layer begins to decompose as the temperature increases (60 °C–90 °C), leading to a loss of interfacial protection. In this condition, the anode becomes directly exposed to the electrolyte, triggering further exothermic reactions, specifically between the intercalated lithium in the anode and the organic solvents in the electrolyte. These reactions generate additional heat, raising the cell temperature further. As the temperature continues to increase, the polymer separator melts, leading to internal short circuits and further accelerating thermal propagation. Ultimately, one of the most significant heat‐generating events occurs, which is the cathode‐electrolyte reaction. This reaction is highly exothermic and releases several times more heat than the earlier anode‐electrolyte reactions, contributing critically to the onset of TR. Here, we compare the thermal behavior of different cell formats and cathode materials using ARC in Table 1.

**TABLE 1:** Comparison of thermal failure of varying cell types, chemistries by ARC.

Type of cell	Cathode	SoC (%)	Total heat release (kJ/kWh)	Methodology	T1, T2, T3 (°C)	Refs.
Prismatic (25 Ah)	NMC	100	N/A	ARC (adiabatic)	90, 124, 259	[75]
Cylindrical (21 700) (4 Ah)	NCA	100	1195	ARC (adiabatic)	86, 227, 608	[76]
Cylindrical (18 650) (2.25 Ah)	NMC	100	3698	Copper slug battery calorimetry (thermally insulated)	N/A, 221, 454	[77]
Cylindrical (18 650) (2.25 Ah)	NMC	50	3902		N/A, 267, 444	
Cylindrical (18 650) (2.25 Ah)	NMC	25	1741		N/A, 238, 341	
Cylindrical (18 650) (2.25 Ah)	NMC	0	667		No TR	
Cylindrical (18 650) (1.5 Ah)	LFP	100	2610		N/A, 243, 311	
Cylindrical (18 650) (1.5 Ah)	LFP	50	1960		N/A, 237, 282	
Cylindrical (18 650) (1.5 Ah)	LFP	25	1375		N/A, 240, 263	
Cylindrical (18 650) (1.5 Ah)	LFP	0	146		No TR	
Cylindrical (18 650) (2.6 Ah)	LCO	100	4460		N/A, 197, 428	
Cylindrical (18 650) (2.6 Ah)	LCO	50	6549		N/A, 227, 425	
Cylindrical (18 650) (2.6 Ah)	LCO	25	6320		N/A, 232, 338	
Cylindrical (18 650) (2.6 Ah)	LCO	0	4183		No TR	
Cylindrical (21 700) (4.8 Ah)	NMC	100	4246		N/A, 179, 753	[78]
Cylindrical (18 650) (1 Ah)	LFP	100	3971	ARC (adiabatic)	N/A, N/A, 271	[79]
Pouch (1.5 Ah)	Li‐S	100	4829	ARC (adiabatic)	70.7, 192.2, 967.6	[80]

Furthermore, the heat released during the exothermic reactions of a LIB cell can also be estimated using temperature data obtained from ARC tests, provided the specific heat capacity (*C*_p_) of the battery is known. Several studies in the literature have outlined methodologies for determining the specific heat capacity of the whole battery cells in the ARC chamber, typically by applying controlled heating power to raise the cell temperature in a stepwise manner [76, 79, 81]. The total energy released is highly dependent on the battery chemistry and the thermal stability of individual cell components. The thermal stability is influenced not only by the intrinsic properties of the active materials but also by the state of charge (SOC) and the pre‐test charging protocol (e.g., charging C‐rate). Safety testing protocols, such as those for thermal abuse and nail penetration, typically require batteries to be tested at 100 % SOC, representing the most thermally unstable condition. Higher SOC generally reduces thermal stability and increases the severity of exothermic reactions. Further investigation is needed to clarify the relationship between (prior) charging C‐rate and the state of safety (SOS), particularly for emerging anode chemistries such as silicon‐based anodes, which exhibit more complex volumetric expansion and fracture behavior than conventional graphite. Understanding these dependencies is critical for designing charging protocols that optimize both performance and safety.

To comply with safety standards, most standard testing protocols rely on temperature‐based metrics rather than direct measurement of energy release. However, batteries with higher specific heat capacity can absorb more thermal energy before exhibiting a noticeable rise in surface temperature. This is particularly relevant for prismatic cells with thick casings, where the delay in surface temperature rise may obscure critical internal thermal events [75]. As a result, internal hotspots, originating from localized exothermic reactions, can develop and initiate TR before any significant change is observed at the surface. Therefore, internal temperature monitoring, for example, using embedded thermocouples or fiber‐optic sensors, becomes crucial, especially for high‐energy‐density cells with high heat capacity [82, 83, 84].

### 3.4 Manufacturing Design

**FIGURE 5:**
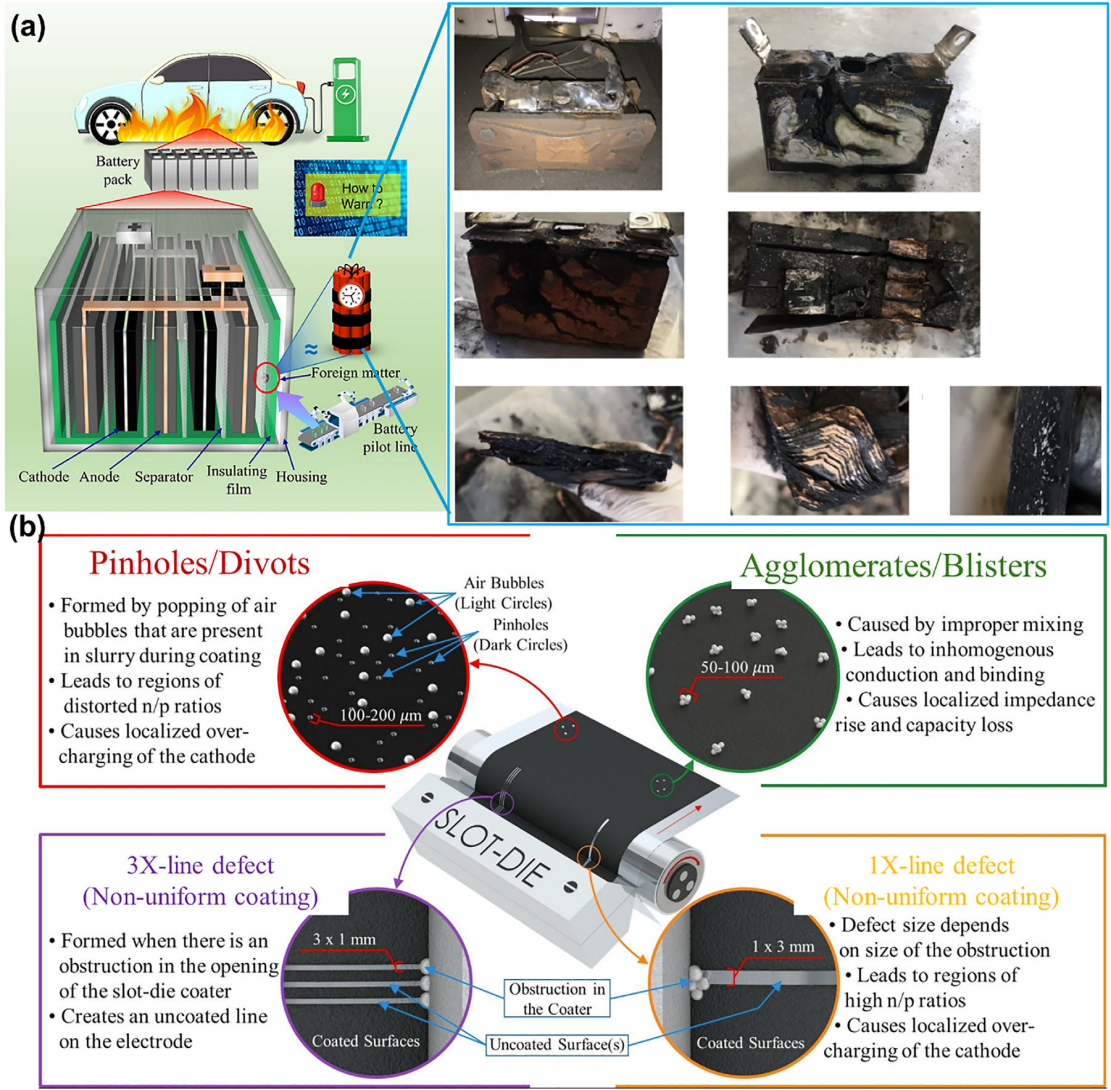
Battery manufacturing flowback design to induce TR during repeated charge/discharge. (a) A case study of foreign particle implantation led to spontaneous combustion events. Reproduced with permission [85], Copyright 2022, Elsevier. (b) non‐uniform electrode coating effect on overcharge, N:P ratio that affects battery thermal behavior. Reproduced with permission [86], Copyright 2018, Elsevier.

TR in LIB occurs when exothermic reactions are initiated by a localized temperature rise, frequently caused by an internal short circuit. Such an ISC can originate from latent manufacturing defects introduced during cell fabrication. Figure 5 summarizes recent findings on how manufacturing‐induced imperfections (including impurities, slurry flowback, and process anomalies) contribute to internal short circuits and TR.

One of the most noticeable defect types arises from the inadvertent incorporation of metallic or foreign particles (i.e., copper debris from the current collector) during coating or calendaring. These contaminants may bridge the anode and cathode, forming localized short circuits. Kong et al. (Figure 5a) reported that foreign particle implantation can lead to spontaneous combustion events, as these particles may puncture the separator under compressive stress, triggering internal shorting that escalates into TR [85].

Another notable manufacturing defect is the non‐uniform coating (see Figure 5b) during electrode fabrication [86]. The coating defects include pinholes or divots, agglomerates or blisters, and line defects (i.e., uncoated current collector surface), etc. Agglomerates formed by improper mixing of the electrode slurry components can lead to localized impedance rise and capacity loss due to poorer conduction and transport within those regions, which increases the voltage loss and heat generation. Pinholes created by air bubbles in the slurry can also cause local imbalances in the negative‐to‐positive (N:P) capacity ratio, which may subsequently cause overcharging or over‐discharging the cell. The line defects formed by obstructions in the coating process are generally the most detrimental. These line defects create uncoated regions in the cathode, distorting the capacity N:P ratio and exposing the current collector to the electrolyte to accelerate the battery thermal degradation. To solve the issue of battery manufacturing defects, advances in computer vision, X‐ray imaging, and AI‐based surface defect detection have been demonstrated as powerful tools to inspect the battery manufacturing process and avoid manufacturing defects to improve battery safety [87, 88].

In this section, we have summarized TR behavior arising from the interplay of mechanical, electrical, thermal, and manufacturing defects. Mechanical deformation and penetration can induce internal short circuits that trigger rapid heat generation, while electrical abuse (overcharge, over‐discharge, and external short circuits) can create the undesired heterogeneous current densities and localized hotspots that accelerate battery thermal failure. In addition, electrode manufacturing defects, including metallic contamination or non‐uniform coating, act as latent failure initiators due to local increases in impedance or temperature or to dendrite proliferation. To quantify the battery TR under the above triggers, ARC can clarify the exothermic reactions and temperature thresholds that lead to TR, thereby revealing strong dependencies on cell chemistry and state of charge. Together, these insights reveal the need for more accurate and timelier in situ monitoring or data‐driven defect detection to predict and mitigate battery TR in next‐generation, high‐energy LIBs.

## 4 Advanced Characterization to Monitor and Predict TR

Early detection of battery thermal failure is essential to give the BMS enough time to take corrective actions and ensure user safety, especially as regulations increasingly require it. The sensitivity of the detection system is vital, and it is particularly important to identify mild reactions at low heating rates that occur before TR. To support the development and validation of early warning systems, it is important to better understand how batteries fail dynamically during TR through advanced laboratory characterization. In this section, we review advanced detection methods, including high‐speed imaging, electrochemical spectroscopy (EIS), acoustic techniques, and sensing methods, which detect or monitor physical, chemical, and electrochemical changes leading up to TR. These can serve as earlier indicators to predict or prevent battery thermal failure.

However, a single technique cannot fully capture the interactions among structural deformation, chemical or gas evolution, temperature rise, interfacial degradation, and electrochemical instability that led to catastrophic failure. Therefore, advanced battery‐failure diagnostics are increasingly adopting multi‐modal, multi‐scale, and data‐driven approaches, reflecting the inherently connected nature of TR. The trend is to combine complementary methods across different lengths and time scales. For example, X‐ray imaging is particularly effective for tracking structural changes. Optical sensing shows superior performance in monitoring local temperature and strain. Acoustic field imaging (AFI) has been used to determine internal heterogeneity and gas formation. Electrochemical impedance spectroscopy (EIS) has been widely used to quantify interfacial and transport behavior. Coupling artificial intelligence and advanced analytics (machine learning) with data processing and prediction from these complex datasets makes this possible and efficient. In this regard, the following subsections review the major advanced techniques above and discuss how they contribute, individually and collectively, to monitoring and predicting thermal runaway.

### 4.1 X‐Ray Imaging

X‐ray computed tomography (CT) has been widely used to visualize the internal 3D structure of materials non‐destructively, both in material and engineering sciences [89]. It has been extended and gradually become an important tool for investigating the thermal failure of LIBs, providing real‐time, non‐destructive nanometer‐scale insights into the internal processes that precede and drive TR. Unlike conventional thermal sensors and voltage monitors, X‐ray imaging enables direct visualization of mechanical, structural, and chemical changes occurring in various battery components. By capturing phenomena such as gas evolution, current‐collector deformation, separator shrinkage, and electrode delamination with high spatial and temporal resolution, X‐ray imaging has enhanced our understanding of battery failure mechanisms and informed the development of safer cell designs.

#### 4.1.1 Short‐Circuit‐Induced Failure in a Single Cell

**FIGURE 6:**
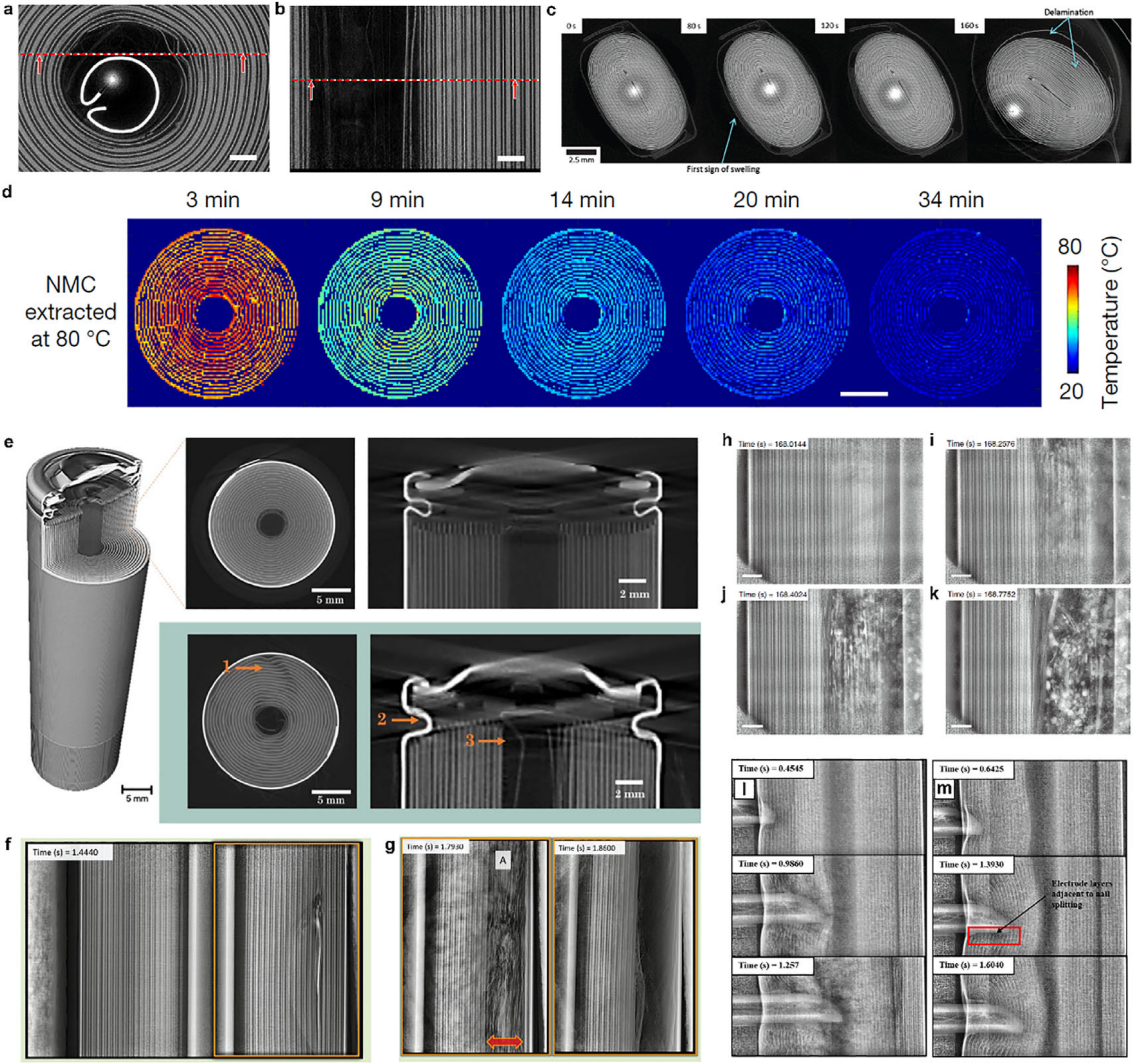
X‐ray imaging of structural and thermal failure mechanisms in LIBs. (a,b) High‐speed X‐ray CT capturing early‐stage TR in 18 650 LIBs, showing (a) enlarged grey‐scale image of the *XY* plane 1 s before TR and (b) enlarged grey‐scale image of the *YZ* plane 1 s before TR. The dotted red lines indicate the through‐plane slice with which the neighboring image is associated. Scale bar, 1 mm. Reproduced with permission under the terms Creative Commons Attribution 4.0 License [28]. Copyright 2015, The author(s), Published by Spring Nature. (c) X‐ray radiographs showing structural evolution during overcharge‐induced TR. Images capture internal short circuits, gas evolution, and separator shrinkage. The marked region highlights electrode deformation. Reproduced with permission under the terms Creative Commons Attribution 3.0 Unported Licence [90]. Copyright 2016, The author(s), Published by RSC. (d) X‐ray diffraction‐CT mapping of internal temperature distribution in an 18650 LIB at 80°C, showing temperature evolution over time as the cell cools. Reproduced with permission [91]. Copyright 2023, Springer Nature. (e) X‐ray macro‐CT results of a commercial 18 650 cell with 3D reconstruction and orthogonal slices in the *XY* and *YZ* planes before TR, and orthogonal slices in the *XY* and *YZ* planes (bottom right two) after TR using ARC, with arrows indicating deformation within the cell architecture. Reproduced with permission under the Creative Commons Attribution 4.0 License [92]. Copyright 2020. The Author(s). Published on behalf of The Electrochemical Society. (f) X‐ray radiograph of a cylindrical LIB during an ISC‐inducedTR, showing electrode layer failure. (g) High‐resolution X‐ray image showing electrode delamination and structural degradation as TR progresses. Both (f,g) are reproduced with permission under Creative Commons Attribution 3.0 Unported Licence [93]. Copyright 2017. The Author(s). Published by RSC. (h–k) Radiographs of TR in Cell 1: (h) *YZ* plane before failure. (i–k) Sequential images show TR starting in the inner layers and spreading outward. Bright spots indicate copper globules. Heating is applied from the right, with a 180° rotation every 0.4 s to maintain uniform temperature. Scale bar, 1 mm. Reproduced with permission under the terms Creative Commons Attribution 4.0 License [28]. Copyright 2015, The author(s), Published by Spring Nature. (l,m): Radiographs of cells undergoing nail penetration. (l) aluminum current collector (CC) + copper CC: TR occurs immediately upon penetration, with electrode cracking visible in the fourth frame. (m) aluminum PCC + copper CC: no TR. However, clear shear stress is observed on the cylindrical electrode. Reproduced with permission under the terms Creative Commons CC‐BY‐NC‐ND License [94]. Copyright 2021, The author(s), Published by Cell Press.

The earliest application of X‐ray imaging to study LIB thermal failure was reported by Finegan et al. in 2015, who employed high‐speed X‐ray radiography and computed tomography (CT) to monitor in situ TR in 18 650 LIBs (Figure 6a,b) [28]. The study demonstrated that gas evolution and electrode delamination occur well before observable external temperature spikes or detectable electrical short‐circuiting. The results indicated that TR is not only a heat‐driven process but also involves the accumulation of internal stresses during failure progression. They revealed that localized gas formation at the electrode–separator interface induces internal pressure imbalances, triggering separator shrinkage and electrode expansion, ultimately leading to catastrophic mechanical failure.

Based on the above findings, Finegan et al. further expanded the use of high‐speed operando X‐ray CT to study overcharge‐induced thermal failure in LiCoO2_2_‐based pouch cells, focusing on gas evolution and separator shrinkage as major contributing factors to failure (Figure 6c) [90]. Their analysis revealed that cells at a high SoC exhibit significantly enhanced gas evolution and separator shrinkage, leading to localized stress concentration and electrode delamination, highlighting the non‐uniform nature of failure propagation. By tracking failure initiation and propagation in real time, they demonstrated that non‐uniform structural weaknesses, particularly at the separator and electrode interfaces, significantly influence thermal failure pathways. These insights provide valuable information on how separator and current collector deformation influence failure progression, which may inform future battery safety strategies.

A more recent study by Heenan et al. utilized X‐ray diffraction‐CT (XRD‐CT) to investigate the internal temperature distribution in cylindrical cells, which can be considered for its role in triggering TR in LIBs (see Figure 6d) [91]. They found that temperature variations within the cell are highly localized, with regions of high electrical resistance exhibiting more rapid heating, which accelerates electrolyte decomposition and failure initiation. This study also revealed that asymmetric temperature distribution within cells, which may be exacerbated with cell ageing. Therefore, non‐uniform thermal distribution can act as an early indicator of failure before catastrophic events occur.

Patel et al. utilized multi‐length‐scale X‐ray CT in combination with ARC to analyze the structural evolution and gas‐phase changes occurring during LIB thermal failure (Figure 6e) [92]. Their study found that electrolyte decomposition leads to the release of CO_2_ and H_2_, with each gas corresponding to different stages of thermal failure. Specifically, CO_2_ formation was observed in the early stages of failure, coinciding with the breakdown of the solid–electrolyte interphase (SEI), while H_2_ generation was primarily attributed to electrolyte decomposition at elevated temperatures. By visualizing these changes in real time, X‐ray imaging provides critical insights into the interplay between structural and chemical transformations that precede catastrophic failure.

Further investigations by Finegan et al. used ultra‐fast synchrotron X‐ray imaging to capture the initiation and propagation of TR, including venting dynamics, in 18 650 cell designs under controlled ISC conditions (Figure 6f,g) [93]. The home‐made ISC device is a four‐layer insert (aluminum pad, thin low‐melting conductive wax, copper puck, copper pad) implanted between the positive current collector and the negative electrode inside the cell. When the wax melts due to the heating, it is wicked into the separator, allowing the aluminum and copper layers to contact and create a controlled, low‐resistance internal short. This on‐demand short reliably initiates TR at a precise, predetermined location. The results showed that uncontrolled pressure buildup can lead to catastrophic ruptures, including sidewall failures and violent cell explosions, rather than controlled venting. Their findings highlighted that ISC‐induced gas accumulation plays a crucial role in accelerating TR, working in conjunction with heat generation and localized reactions. Importantly, they demonstrated that additional venting mechanisms can significantly mitigate the risk of explosive failure by alleviating internal pressure buildup.

To further investigate the mechanical factors contributing to TR, Pham et al. conducted nail penetration tests to induce localized short circuits (Figure 6l,m) [94]. Comparing conventional aluminum and copper current collectors with polymer‐based replacements, the results demonstrated that these polymer‐substrate current collectors effectively isolated internal short circuits by physically retracting from the heating region, thereby preventing the escalation of thermal failure. Cells using these current collectors exhibited a significantly lower risk of TR under abuse conditions, with aluminum‐coated polymer current collectors achieving 100% success in preventing TR. In addition to the above nail penetration, an ISC device was designed and placed in a cylindrical cell to generate an internal electrical short [93]. It would trigger rapid gas generation and pressure rise due to the extremely high current and temperature observed. Following this, efficient, symmetric venting can limit mechanical rupture, whereas obstruction or asymmetric flow leads to uncontrolled ejection and a higher risk of affecting adjacent cells. These findings reveal the importance of vent design in slowing or preventing thermal propagation.

#### 4.1.2 Cell‐to‐Cell TR Propagation

**FIGURE 7:**
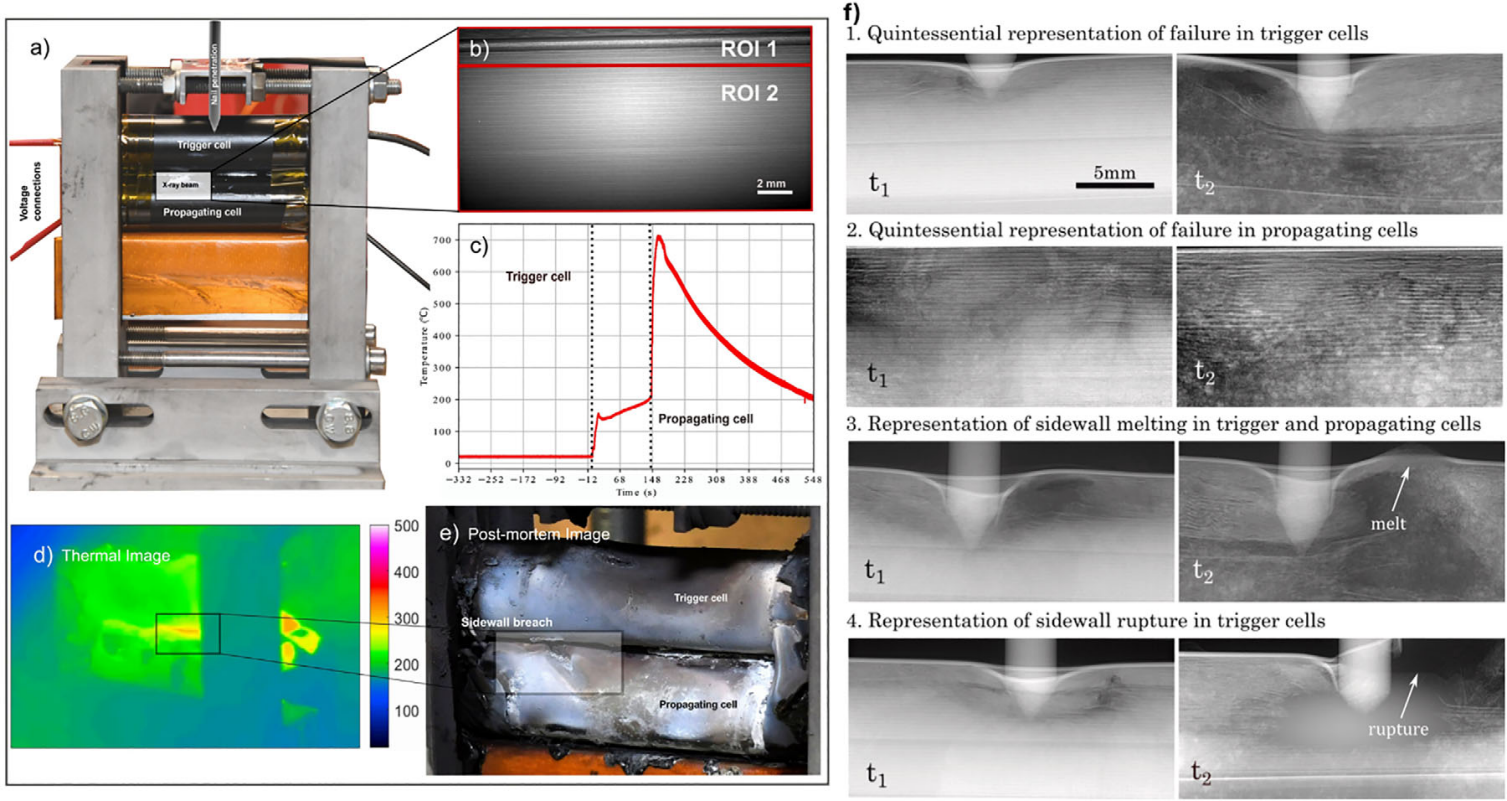
X‐ray‐assisted observations of the cell‐to‐cell TR propagation: (a–e) in situ visualization of a sidewall breach during TR driven by cell‐to‐cell propagation in Figure 7a–e. Reproduced with permission [95], Copyright 2023, Elsevier. (f) propagation tests to observe TR dissipation under series and parallel configurations. Reproduced with permission under the terms Creative Commons Attribution 4.0 License [97]. Copyright 2024, The author(s), Published by Elsevier.

TR propagation between cells remains one of the most hazardous failure modes in multi‐cell LIB systems, as localized failure can cascade through modules or packs within seconds. To achieve TR monitoring, high‐speed synchrotron X‐ray imaging has revealed the internal mechanical and thermal dynamics that govern whether and how propagation occurs. In this regard, Fransson et al. have made the first in situ visualization of a sidewall breach during TR driven by cell‐to‐cell propagation in Figure 7a–e [95]. Using the spatial‐temporal mapping (i.e., the Gabor Filtering technique), Paul's team tracked electrode structure deformation at the onset of TR [96, 97]. They also quantified the rate of electrode delamination, directly linked breach sites to localized thermal hotspots in the jellyroll, and demonstrated that heat transfer through cell contact points initiates internal combustion before the casing ruptures.

Following this, Fransson et al. also conducted 32 controlled propagation tests to compare NCA (P42A) and NMC (M50) chemistries under series and parallel configurations, as shown in Figure 7f [97]. Propagation occurred in 56 % of cases, with a minimum ∼150°C in the neighboring cell required for initiation. Series‐connected cells tended to reach higher propagation‐cell temperatures and exhibit faster internal TR evolution, which is attributed to higher internal resistance and absence of current dumping. High heat rates (>0.4 °C s^−1^) in the propagation cell were statistically associated with propagation events.

#### 4.1.3 Integrating X‐Ray Imaging With Multimodal Diagnostics

Despite its significant benefits, X‐ray imaging alone cannot serve as a comprehensive early‐warning system for battery failure, as it mainly detects structural changes that have already occurred. To improve early‐stage failure detection in labs, researchers are combining X‐ray imaging with other techniques, such as neutron imaging, electrochemical impedance spectroscopy (EIS), and machine‐learning‐based diagnostics. Neutron imaging, in particular, allows tracking of low‐density components inside a battery, a feature that X‐ray imaging alone cannot provide. Together, neutron and X‐ray tomography enable effective assessment of lithium transport and distribution in all‐solid‐state batteries, even at low current densities.

Additionally, AI‐driven data analysis is being developed to automate the interpretation of vast X‐ray datasets, enhancing predictive accuracy and supporting real‐time risk evaluation [98]. In this regard, recent advances in X‐ray imaging have enabled the quantitative extraction of thermal degradation kinetics from time‐resolved three‐dimensional datasets. The kinetic parameters can be directly obtained, including crack initiation and propagation rates, particle degradation metrics, and gas evolution kinetics [99, 100, 101, 102, 103, 104, 105, 106, 107]. For instance, Hao et al. demonstrated, through time‐resolved synchrotron X‐ray tomography, that crack propagation in solid electrolytes exhibits a rapid initial growth followed by a decelerating trend, while the evolution of electrode thickness and lithium filling ratios within cracks can be quantitatively tracked throughout cycling [99]. By further integrating image‐derived morphologies with multiphysics modelling, the evolution of mechanical stress and electric potential fields can be reconstructed, revealing that the initial release of strain energy upon first penetration acts as a dominant driving force, followed by coupled electro‐chemo‐mechanical processes that govern subsequent degradation. Parks et al. achieved statistically robust quantification of cracking across more than 7000 particles [103]. The analysis reveals a non‐linear dependence of cracking on applied potential, with measurable fracture occurring at relatively low voltages. This indicates that degradation at elevated voltages is not primarily governed by mechanical cracking but is driven by electrochemical processes, including electrolyte reactions and oxygen loss. The above quantitative parameters (i.e., cracks, stress) from X‐ray imaging are particularly valuable because they provide direct links between observed structural evolution and the underlying electrochemical, thermal, and mechanical driving forces. Combining X‐ray imaging and machine learning would be a powerful tool for predicting battery thermal degradation or thermal runaway.

### 4.2 Optical Sensing Techniques

**FIGURE 8:**
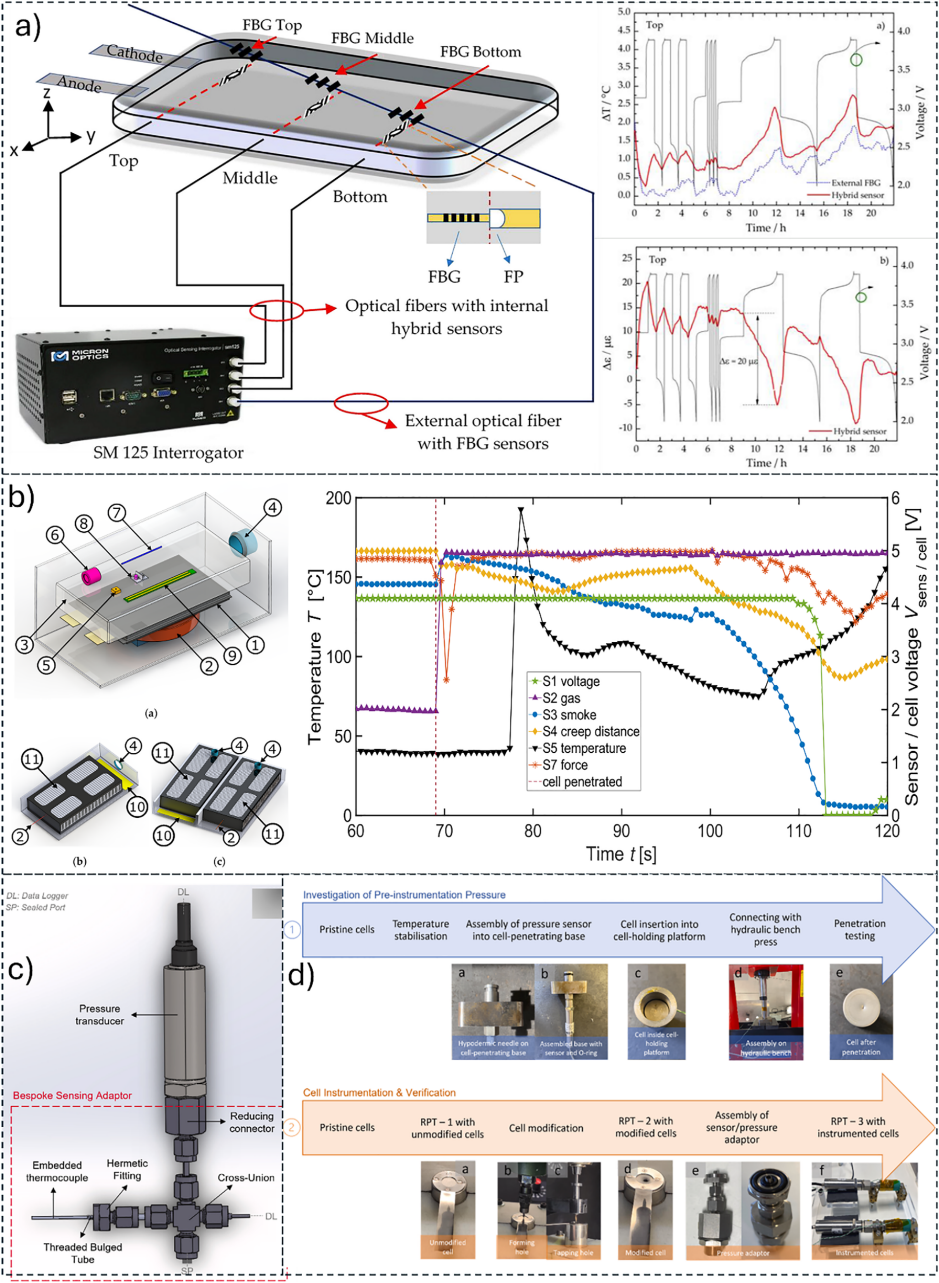
Applications of sensing technology to monitor internal temperature, pressure, stress, and smoke to indicate battery TR: (a) optical fibers with internal hybrid sensors (temperature and pressure) to monitor battery temperature and pressure [109]. (b)Three different tests for tracking the cell pressure, gas, temperature, and detecting smoke, and creep distance. Reproduced with permission under the terms Creative Commons Attribution License [112]. Copyright 2018, The author(s), Published by MDPI. (c,d) Internal pressure and temperature sensing design before or during battery TR. Reproduced with permission under the terms Creative Commons Attribution License [120, 121]. Copyright 2023 [120]. and 2024 [121]. The Authors. Published by Elsevier.

Accurate monitoring of internal battery temperature is fundamental for TR prediction and to mitigate battery thermal failure. Normally, the traditional surface‐mounted thermocouples offer limited responsiveness (i.e., time or spatial lag). However, the embedded micro‐sensors and fiber‐optic thermometers could enable the real‐time tracking of internal thermal gradients. It has been demonstrated that integrated sensors can identify temperature rise events significantly earlier than external parameter indications [108]. A hybrid sensing network comprising fiber Bragg gratings and Fabry–Perot cavities has been proposed for the simultaneous monitoring of strain and temperature, as shown in Figure 8a [109]. The designed network was embedded within a pouch cell to monitor internal variations in strain and temperature at different electrode positions.

Internal mechanical stress is a potent early indicator of material degradation when using the sensing technique. Operando stress‐sensing technologies, such as piezoresistive and piezoelectric sensors, would translate stress variations into measurable electrical outputs. Benefiting from this, Shen et al. provided a comprehensive overview of thermal and mechanical stress‐sensing methodologies in LIBs [110]. However, issues such as high environmental interference, complex parameter separation, the fragility of optical fibers, and low adhesion compatibility with batteries still need to be overcome to enable accurate battery stress sensing. Additionally, Wang et al. emphasized the importance of smart sensors capable of simultaneously tracking mechanical and thermal signatures​ [111]. Fast‐response stress monitoring in Figure 8b was achieved by Koch et al. to enable early intervention in large‐scale traction battery systems, and it shows strong scalability potential for electric vehicle applications [112]. Apart from the above pressure detection, the work also includes multiple‐parameter sensing (e.g., smoke, internal temperature, internal stress, etc.), designed to reveal battery degradation and thermal behavior as early indicators to avoid battery TR.

Swelling, resulting from gas evolution and material expansion during battery cycling/degradation, provides a direct physical indicator of internal failure processes. Sponge‐based piezoresistive sensors were demonstrated to detect swelling‐related changes that correlate with state‐of‐charge and battery failure (lithium plating and TR) [113]. Furthermore, stretchable carbon nanotube dilatometers, as shown by Wang et al., enable real‐time in situ monitoring of minor volume changes by integrating a stretchable CNT‐based strain sensor on the surface of the LIB [114]. The CNTs formed a percolation network on the elastomeric substrate, exhibiting reversible electromechanical stretchability and a high strain response (gauge factor (GF) of ∼50–373). This high GF value indicates superior, reversible stretchability under repeated and pronounced volumetric expansion. The high‐strain response would allow the sensor to accurately track the swelling behavior of LIBs, arising from both regular charge/discharge processes and irregular thermomechanical stresses that occur before TR. Additionally, Cai et al. demonstrated the use of swelling metrics to diagnose internal shorts well before TR [].

Gas sensing is another step toward understanding the thermal degradation of the battery before TR occurs. Gas generation is a byproduct of electrolyte decomposition and electrode active material breakdown during battery failure. To achieve the gas chemistry monitoring, the optical fiber‐based gas sensors have emerged as leading candidates for early TR detection due to their small form factor and immunity to electromagnetic interference [116, 117] Jin et al. captured micro‐scale lithium dendrite formation by detecting hydrogen gas evolution, thus providing a methodology to avoid the battery internal short circuits and thermal failure​ [118]. Meanwhile, the gas sensors were developed to detect volatile organic compounds (VOCs) and specific TR precursor gases, such as CO_2_ and HF [111, 119]. These advancements not only enhance sensitivity but also provide chemical specificity to failure detection [116, 117]. Meanwhile, the gas sensors were developed to detect VOCs and specific TR precursor gases, such as CO_2_ and HF [111, 119]. These advancements further enhance sensitivity and provide chemical and pressure value specificity to failure detection in Figure 8c,d [120, 121].

Early detection of TR using advanced sensing technologies would enable safer, more resilient battery energy storage. Despite significant advances, several challenges may need to be addressed to enable accurate, fast, and stable physical (temperature, pressure, expansion, stress), chemical (gas), and electrochemical (voltage, current, etc.) diagnosis during long‐term cycling (Table 2). First, embedding sensors inside cells may alter the internal architecture and introduce new failure pathways, and the durability of embedded systems under repeated cycling and thermal stress remains understudied. Hybrid sensing strategies, combining thermal, mechanical, and chemical data, are necessary to characterize battery state‐of‐safety and subsequently achieve reliable early warning, and sensors that enable this correlative diagnosis should be prioritized. In addition, signal processing is still a bottleneck. The sensor data needs advanced filtering, machine learning, and fusion algorithms to extract actionable insights. Finally, the cost considerations and standardization are hurdles for scaling these innovations into BMS. Therefore, continued interdisciplinary research and development will be essential to address current limitations and bring these systems into mainstream adoption.

**TABLE 2:** Comparison of the sensing technology in battery storage.

Sensing technology	Advantages	Limitations
Temperature sensing	‐ Direct measurement of thermal hotspots ‐ Fast response with embedded sensors ‐ Well‐integrated into existing BMS architectures	‐ May miss localized failures if the spatial resolution is low ‐ Invasive installation can affect the battery structure ‐ Thermal lag if only using external sensors
Stress sensing	‐ Detects mechanical failures before heat generation ‐ Sensitive to early‐stage internal shorts or dendrite growth ‐ Non‐destructive and continuous monitoring is possible	‐ Signal interpretation can be complex (stress vs. thermal effects) ‐ Sensor calibration is needed for different cell chemistries ‐ Sensor fatigue over long cycles
Swelling sensing	‐ Highly sensitive to gas evolution and electrolyte breakdown ‐ Swelling is detectable before major heating ‐ Enables mechanical health monitoring	‐ Small swellings at early stages can be difficult to detect without high‐precision sensors ‐ Risk of mechanical integration challenges ‐ External swelling sensors may be less accurate than internal ones
Gas sensing	‐ Direct chemical evidence of degradation ‐ High sensitivity to TR precursor gases (H_2_, CO_2_, HF) ‐ Enables classification of failure modes (shorts, overheating, etc.)	‐ Installation requires careful sealing and positioning ‐ Cross‐sensitivity to ambient gases

### 4.3 Acoustic Field Imaging

Monitoring early‐stage indicators of TR, such as structural degradation and gas generation, is essential for developing safer batteries and predictive diagnostic tools. Among emerging diagnostic methods, Acoustic field imaging (AFI), based on ultrasonic wave propagation through battery materials, offers a powerful, non‐destructive approach to monitoring the internal state of batteries. Generally, the AFI sends acoustic waves into a battery and analyzes the patterns of transmission and reflection. This pattern evolution results from changes in wave speed due to internal structural changes (such as electrolyte depletion, gas pocket formation, and mechanical damage) within the cell.

**FIGURE 9:**
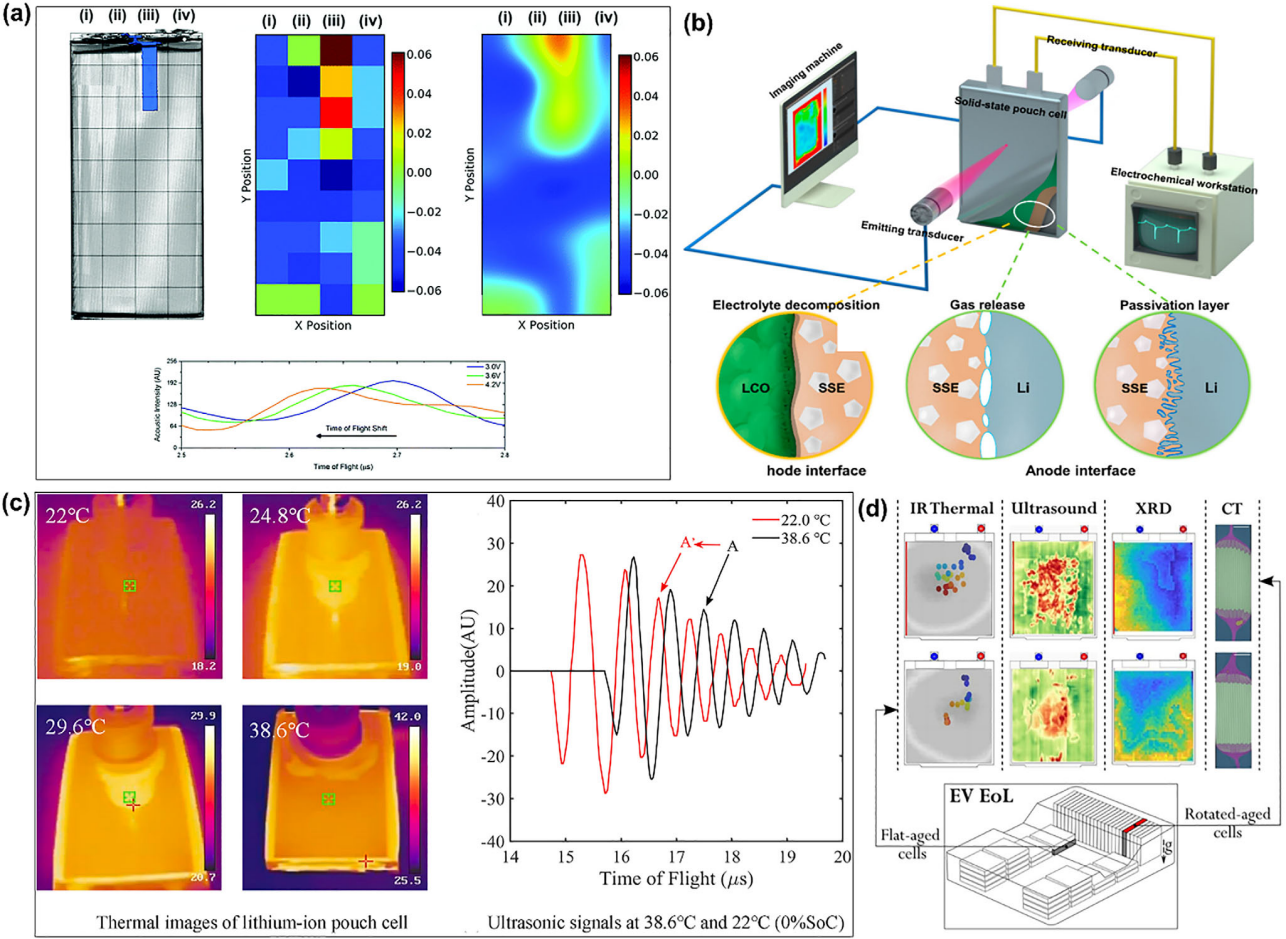
Acoustic‐field imaging to detect the battery temperature and structure evolution as an early monitoring to prevent battery TR: (a) spatially resolved mapping through ultrasonic ToF analysis at 36 distinct locations on a mobile phone battery. Reproduced with permission under the terms Creative Commons Attribution 3.0 Unported Licence. Copyright 2019, The author(s), Published by RSC [123]. (b) Application for the acoustic field to track the battery parameters (gas evolution) before TR. Reproduced with permission, copyright 2022, American Chemical Society [124] (c) Case of battery temperature variations influencing ultrasonic reflection signals. Reproduced with permission, copyright 2022, Elsevier [125]. A combination of varying advanced techniques (IR thermal, acoustic, XRD, and CT) to inform battery degradation and TR (d) [128]. Reproduced with permission under the terms Creative Commons Attribution 4.0. Copyright 2023, The author(s), Published by Elsevier.

As the originators of the application of AFI in the battery field, Steingart et al. pioneered this technique to probe internal mechanical, structural, and electrochemical changes in sealed battery cells, correlating acoustic wave propagation (time of flight, amplitude, attenuation) with state‐of‐charge, state‐of‐health, and degradation phenomena [122]. However, early applications of ultrasound in battery diagnostics primarily relied on fixed‐point measurements, thus limiting the ability to detect heterogeneity across the full area of a LIB. A significant advancement was made by Robinson et al., who first demonstrated spatially resolved mapping using ultrasonic time‐of‐flight (TOF) analysis at 36 distinct locations on a pouch cell, thereby identifying inhomogeneities within the cell, as shown in Figure 9a [123]. Their study showed that defect depth could be determined, and the variations in signal amplitude correlated with the state of lithiation of the electrodes. Following this, we will discuss how recent acoustic technique developments can detect internal temperature rise and gas formation.

#### 4.3.1 Detecting Gas Formation

Internal gas evolution in LIBs is one of the earliest indicators of cell degradation or thermal failure. It not only impedes Li^+^ transport between the anode and cathode but also decreases battery capacity and performance. Therefore, timely gas detection is necessary for early hazard identification. Conventional diagnostic approaches (i.e., mass spectrometry, gas chromatography, or pressure sensors) provide limited temporal resolution and are typically invasive. Electrochemical testing (EIS, voltage–capacity analysis) can only indirectly inform us about gas formation and is often obscured by concurrent interfacial reactions. Therefore, technology that focuses on dynamic, non‐invasive monitoring of gas generation during cycling is highly desirable.

To achieve this, acoustic or ultrasonic sensing, in contrast, provides direct, non‐invasive, and highly sensitive detection of internal gas formation. The detection sensitivity arises from the large acoustic impedance mismatch (i.e., varying dissipation routes and the increasing number of encountered interfaces) between the gas phases and the surrounding solid–liquid battery components [122]. Therefore, it can generate measurable changes in acoustic transmission and reflection characteristics, enabling detection of targets larger than 0.6 mm, such as potential gas bubbles or voids. These gas bubbles/voids scatter and attenuate acoustic waves far more strongly than solid or liquid regions, which leads to distinct amplitude damping, phase shifts, and time‐of‐flight variations in output signals. As a result, it can detect the onset of gas generation and internal defects before observable cell swelling or pressure build‐up, thus enabling early‐failure identification, as shown in Figure 9b [124]. Furthermore, as ultrasonic waves readily penetrate metal and polymer casings, the technique allows in operando gas monitoring under realistic cycling or abuse conditions without breaching cell integrity. Beyond early detection, ultrasonic monitoring could also be applied to thermal‐failure and safety studies by tracking the buildup and evolution of internal gas formation before the TR occurs. These acoustic signatures correlate strongly with high‐speed X‐ray observations (i.e., electrode delamination), thereby providing a time‐resolved link between internal gas evolution, structural changes, and the thermal‐mechanical escalation leading to failure.

#### 4.3.2 Internal Temperature Monitoring Before TR

Given its advantages in real‐time monitoring, high precision, non‐invasiveness, and non‐destructivity, ultrasonic technology has been increasingly explored for detecting changes in cell internal structure and temperature. Numerous efforts have demonstrated the feasibility of ultrasonic methods for the early TR detection. Ke et al. investigated how variations in battery temperature affect ultrasonic reflection signals. The ToF of acoustic waves increases linearly with temperature and state of charge (SOC) in Figure 9c [125]. Popp et al. employed ultrasonic signals to monitor internal temperature changes in batteries [126]. These studies confirm the viability of ultrasonic sensing for tracking internal thermal changes. Additionally, McGee et al. used transmission waves to characterize internal alterations (temperature and structure) before TR, demonstrating that variations in ToF can serve as indicators of thermal damage within the cell [127]. These findings demonstrated the high sensitivity of ultrasonic techniques to internal structural and thermal changes, indicating their potential as an effective strategy for the early warning and prevention of battery TR.

#### 4.3.3 Ultrasonic Signal Processing and Interpretation

Despite the promising prospects of ultrasonic technology for battery inspection, several technical challenges in data accurate processing remain.

First, various parameters, including temperature changes, gas generation, stress, and layer separation, can influence the TOF and peak amplitude. Therefore, accurate interpretation or decoupling cannot rely on a single peak or scalar descriptor. To address this, multi‐physics models provide a mechanistic framework to differentiate waveform changes caused by temperature‐dependent variations in sound velocity and modulus from those resulting from gas pockets, delamination, or stresses. In the 1D layered‐medium model developed by Copley et al., the waveform is directly related to layer thickness, density, elastic modulus, and acoustic impedance, explaining why similarly sized cells can produce different signals and why feature selection must be cell‐ and waveform‐specific [129]. Ma et al. developed a transfer‐matrix model for the periodic electrode stack and derived the dispersion relation using the Floquet–Bloch theorem, showing that commercial pouch cells exhibit a Bragg bandgap and that ultrasonic reflection, transmission, and waveform distortion are strongly frequency‐dependent [130]. They also created a finite‐element model in COMSOL Multiphysics to simulate wave propagation and scattering within the layered cell, including acoustic coupling at the water–cell interface. This model confirmed the dispersion behavior predicted by the analytical model, compared responses under pass‐band, near‐bandgap, and bandgap excitations, and identified frequency ranges more sensitive to internal structural changes. These findings provide a basis for selecting excitation frequencies and interpreting waveform variations that cannot be explained by peak tracking alone. The same transfer‐matrix/FEM approach has also been used to segment reflected signals into front‐surface reflection, rear‐surface reflection, and internal scattering components, enabling structural changes to be associated with specific waveform features rather than treated as a single undifferentiated trace [131].

In addition, the structural and material diversity across LIB types poses a significant barrier. Variations in geometric design and composition make it difficult to identify a single set of ultrasonic parameters that is universally applicable. Currently, ultrasonic methods are most effective for pouch cells, whereas cylindrical batteries pose additional challenges. The presence of gaps between the jelly roll and the external casing in cylindrical cells reduces the penetration efficiency of low‐energy ultrasonic waves, limiting their diagnostic capability. Furthermore, possible acoustic artefacts can arise from sensor coupling to the curved surface and from the propagation of acoustic waves around the metal cylinder.

Third, data management is another major hurdle. A single battery scan can yield tens of thousands of waveform data points or data noise during testing, making the efficient extraction of meaningful features a non‐trivial task and often requiring dimensionality reduction or data‐driven analysis [132]. This challenge can be mitigated by machine‐learning‐based holistic waveform analysis, in which the full ultrasonic response is processed and the most informative features are selected statistically, thereby reducing dimensionality and avoiding manual, potentially arbitrary peak selection [133]. Beyond feature extraction from high‐dimensional data, machine learning has also been applied to signal recovery in cases where waveform interpretation is hindered by echo overlap. Chapon et al. showed that convolutional neural network (CNN)‐based deconvolution, trained on simulated data, can separate strongly overlapping echoes and recover reflector arrival times without increasing transducer frequency, thereby improving axial resolution while preserving penetration depth [18, 19, 20, 27]. This is particularly pertinent to AFI, in which reflections from current collectors, internal interfaces, voids, and gas pockets may overlap within the same temporal window. Deconvolution therefore improves ToF extraction, reduces ambiguity in reflector assignment, and makes subsequent feature tracking more reliable. Its role is best understood as a front‐end signal recovery step that enables more accurate physical interpretation of layered battery waveforms.

Overcoming these obstacles will require continuous advancements in ultrasonic technologies, including improved penetration depth, enhanced signal processing algorithms, and developing robust calibration methods that account for both environmental conditions and battery‐specific variability. Given both mechanical changes and temperature influence acoustic ToF/amplitude, decoupling these two causes remains challenging. Nevertheless, machine‐learning‐assisted acoustic analysis and advanced coupling strategies (IR, ultrasound, CT, etc.) are actively being developed to overcome these limitations, as shown in Figure 9d [128].

### 4.4 Electrochemical Impedance Spectroscopy

Electrochemical impedance spectroscopy (EIS) has emerged as a promising alternative, offering real‐time, non‐invasive insights into internal electrochemical changes that precede catastrophic failure [21]. It detects frequency‐dependent impedance variations, revealing interfacial instability, shifts in charge transfer resistance, and lithium‐ion transport degradation, establishing it as a predictive battery health‐monitoring tool [134, 135].

**FIGURE 10:**
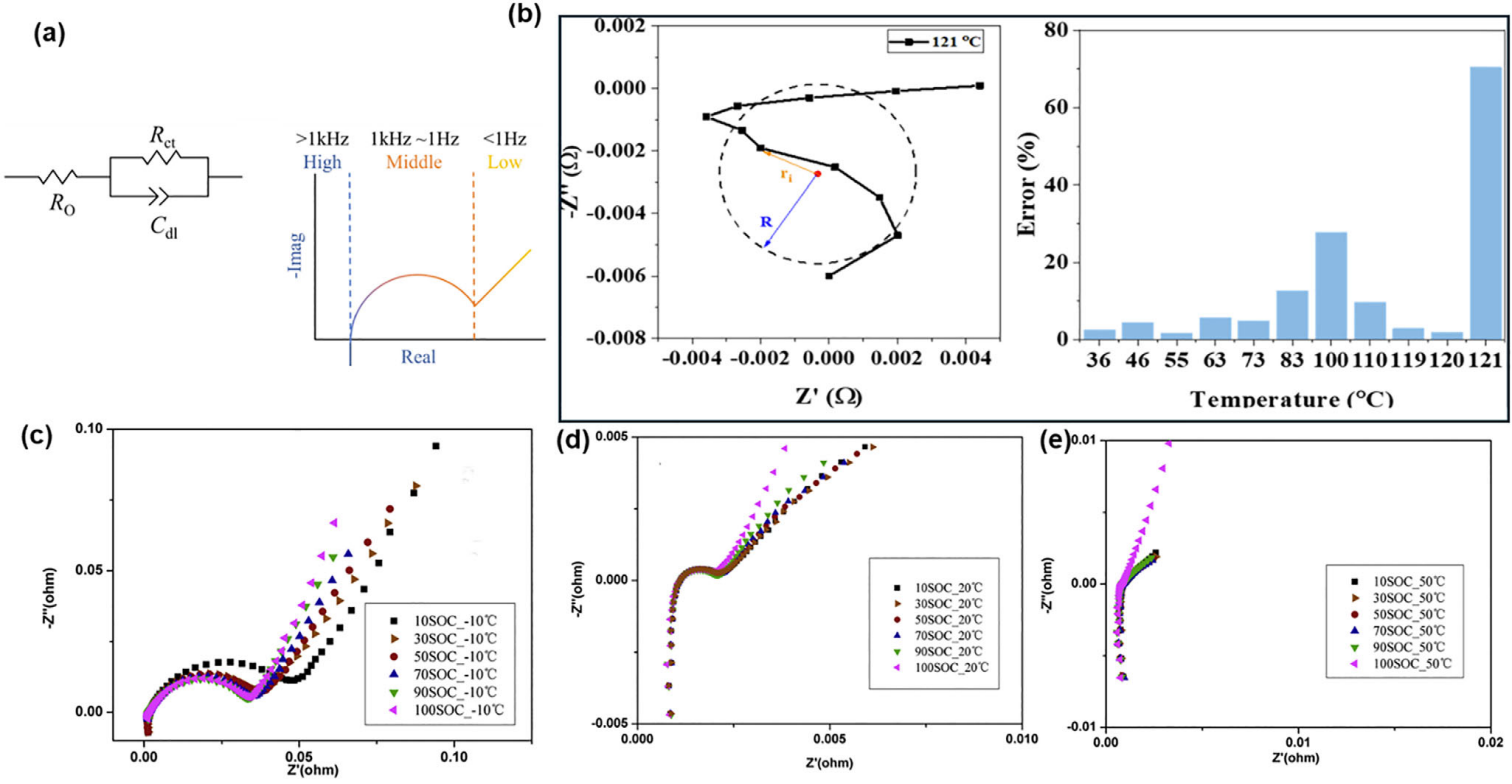
EIS spectrum for monitoring cell temperature evolution: (a) Nyquist plot of LIB and a common equivalent circuit model. Reproduced with permission [135], Copyright 2024, Elsevier. (b) EIS) for TR detection in LIBs. It includes a Nyquist plot at 121°C, showing impedance changes with increasing temperature and fitting errors at different temperatures, highlighting rising errors in extreme conditions. Reproduced with permission [134], Copyright 2021, The Electrochemical Society (“ECS”). (c–e) Temperature‐dependent impedance spectra at various temperatures, illustrating shifts in characteristic frequencies under thermal stress. These trends support EIS as an early diagnostic tool. Reproduced with permission [136], Copyright 2014, Elsevier. B.V.

EIS‐based early warning mechanisms have been systematically explored to correlate impedance shifts with the onset of TR. Dong et al. established a two‐stage warning system (shown in Figure 10), identifying intermediate‐frequency (∼30–500 Hz) phase angle shifts, particularly at 31.62 Hz in charge‐transfer resistance, as an early indicator of internal temperature rise, enabling an early warning signal for battery thermal degradation before thermal escalation [134]. As degradation progresses, low‐frequency (<10 Hz) deviations indicate cell deformation, while high‐frequency (>10 kHz) variations indicate separator degradation and the risk of internal short circuits. Li et al. further improved this methodology by demonstrating a three‐stage early warning system under thermal abuse conditions, where low‐frequency impedance increases correspond to gas formation and electrolyte decomposition, mid‐frequency fluctuations track SEI breakdown and lithium plating, and high‐frequency deviations reflect mechanical stress and separator failure [135]. By integrating real‐time EIS with an ARC‐EIS system, they quantified warning thresholds using the ‘RReliefF’ machine learning algorithm and extracted key impedance features for predictive safety optimization.

Beyond early warning, EIS has been explored for internal temperature monitoring to address the limitations of conventional thermal sensors. Zhu et al. established a strong correlation among phase shift, impedance‐magnitude variations, and internal temperature, demonstrating EIS as a viable alternative to embedded thermocouples [136]. They developed an electrochemical impedance matrix analysis that revealed an intrinsic relationship between impedance phase changes and internal temperature. Spinner et al. expanded on this by correlating imaginary impedance (−*Z*_imag_) at 300 Hz with internal temperature, showing that this correlation remains independent of the state of charge [137]. They applied an Arrhenius‐type model to determine an activation energy of 0.13 eV for SEI ionic conductivity, which provides a key metric for tracking thermal behavior. Their findings demonstrated that single‐point EIS diagnostics enable near‐instantaneous temperature tracking, overcoming the limitations of surface‐mounted sensors, which often fail to detect temperature gradients within the cell.

To achieve more accurate and faster battery thermal failure monitoring, EIS, combined with advances in frequency‐domain analysis and AI‐driven models, is becoming a key tool for next‐generation battery safety. Machine learning enhances impedance diagnostics by refining early‐warning thresholds and reducing false positives. In this regard, integrating EIS with structural imaging further improves failure detection, enabling a shift from reactive safety measures to proactive battery health monitoring.

### 4.5 Real‐World Implications

Whilst these advanced laboratory characterization tools (X‐ray imaging, sensing, AFI, and EIS) have significantly deepened our understanding of TR in LIBs, their ultimate impact lies in their translation into real‐world applications. In Table 3, we consider the translational potential of the techniques. Their capability for field deployment, on‐board monitoring, with the associated volume and cost implications, has been taken into consideration.

**TABLE 3:** The application potential of advanced laboratory characterizations in the real world.

Characterization	Industrial translation potential	Deployment status
X‐ray imaging of TR	High‐resolution imaging of failure modes, cell deformation, and venting behavior.	Research‐grade: too expensive for in‐field use. Primarily used for validation of cell/pack design and failure investigations.
Optical sensors	Real‐time monitoring of internal temperature, pressure, and swelling using fibre optics.	Field‐deployable: embedded fibre Bragg gratings, stretchable CNT sensors, and Fabry–Perot cavities are being integrated into cells and packs.
Acoustic imaging	Detection of internal structure changes, gas generation, and temperature via ultrasound.	Semi‐deployable: external ultrasonic probes and adhesive transducers can monitor commercial cells without altering pack structure.
EIS	Real‐time state‐of‐health (SoH) and thermal behavior monitoring via impedance trends.	Emerging commercial adoption: in‐cell impedance tracking is being explored for real‐time diagnostics.

Emerging diagnostic and characterization tools must move beyond experimental lab scales and be incorporated into deployable monitoring systems that interact with BMS. Due to equipment complexity, size, and cost, X‐ray imaging remains confined to research and metrology applications and is clearly not suited to on‐board diagnostics. However, its role in validating failure mechanisms and informing battery design strategies is highly relevant to improving battery design and certification. For example, X‐ray‐assisted identification of venting failures and sidewall breaches has directly informed pack architecture modifications, such as enhancing vent symmetry and adding internal heat sinks. These findings serve as a foundation for downstream sensor deployment, identifying where in the battery the earliest physical indicators of failure arise.

In addition, optical sensors can track internal temperature, pressure, and mechanical strain within pouch and cylindrical cells. Nevertheless, the embedded and invasive designs may alter the electrical and chemical environment of the battery pack. To minimize the above impact on the BMS, a sensor with multiple uses to simultaneously detect battery physical and chemical changes is desired.

Then, AFI is non‐invasive and has been demonstrated to be widely compatible with pouch or prismatic cell formats. With increasing sophistication in data processing, it is poised for application in electric vehicle and aerospace battery packs, where early detection of internal anomalies is important, and can also be widely applied in manufacturing QC and diagnostics.

BMS‐compatible hardware should support real‐time impedance measurement using pulse or frequency‐domain methods, allowing EIS‐derived metrics to inform state‐of‐health (SoH), state‐of‐safety (SoS), and failure prediction in operational battery systems.

Despite the above hurdles, the emerging convergence of advanced sensing, BMS algorithms, and machine learning still marks a significant step toward safer, smarter batteries. The transition of these techniques from the lab to industrial application is underway. As the above diagnostic technologies mature and become cost‐effective, their integration will be critical for meeting the growing energy demands of electrified transport and renewable storage without compromising safety.

## 5 Summary and Outlook

The application of LIBs across transport, grid storage, and consumer electronics is accelerating the green energy transition to achieve global decarbonization. However, safety concerns (especially the rare but catastrophic phenomenon of TR) continue to present a major barrier to public trust, regulatory approval, and technological scalability. TR is a complex, multi‐stage phenomenon governed by tightly coupled electrochemical, thermal, and mechanical processes. The introduction (Section 1) highlights the urgent need for a comprehensive understanding of TR to ensure the safe expansion of electrified energy storage. Then, Section 2 elucidates the sequential evolution of TR, from SEI breakdown and separator shrinkage to electrode–electrolyte reactions and oxygen release. This section also discusses how localized heat generation, gas formation, and mechanical deformation interact to accelerate failure and propagate between cells. Section 3 further summarizes TR trigger modes under different abuse scenarios: mechanical (indentation, compression, and nail penetration), electrical (overcharge, over‐discharge, and external short circuits), thermal (accelerating‐rate calorimetry), and manufacturing‐related defects. These investigations demonstrate that the onset and severity of TR depend strongly on state of charge, cell geometry, electrode thickness, and defect morphology. Building on these insights, we summarize recent advances in characterization techniques and discuss their potential translation to real‐world applications in Section 4:

First, X‐ray imaging, particularly high‐speed radiography and tomography, has provided valuable insights into the physical structure and chemical gas dynamics, enabling prediction or observation of TR. Numerous synchrotron X‐ray imaging studies have captured phenomena such as electrode delamination, gas formation, and structural deformation that precede catastrophic failure. However, due to equipment complexity, size, and cost, X‐ray imaging remains confined to research applications as opposed to field‐deployable safety diagnosis. However, its role in validating failure mechanisms and informing design strategies is indispensable.

For optical sensing, several lab‐developed technologies have made measurable progress toward field‐deployable solutions. Fiber‐optic sensors, such as fiber Bragg gratings and Fabry–Perot cavities, have demonstrated the capability to monitor in situ internal temperature, pressure, and mechanical strain within pouch and cylindrical cells. These sensors can be embedded into the cell structure during manufacturing, offering real‐time monitoring of internal gradients and physical deformations. Similarly, stretchable piezoresistive films and carbon nanotube‐based dilatometers can monitor cell swelling, an early physical indicator of gas evolution, providing a critical window for intervention before TR onset in the pilot‐scale battery packs. However, due to its invasive design, the current Li^+^ path may be altered. Miniaturization of sensors capable of simultaneous correlative parameterization (pressure, temperature, strain, etc.) would be highly valuable. Whilst further cost reductions are also needed, the incorporation of sensors in a small number of ‘exemplar’ cells within packs may provide a pathway to cost‐effective adoption.

Third, AFI offers another promising technology for TR detection, especially in large‐format cells where internal structural changes may not be externally visible. Ultrasonic transducers, adhered to the battery, can detect subtle changes in acoustic wave velocity and reflection caused by gas generation, layer delamination, and temperature shifts. While their signal interpretation remains complex, often requiring machine‐learning‐assisted analysis, it demonstrates significant potential for electric vehicle and aerospace battery packs.

Last, EIS bridges the gap between laboratory characterization and BMS‐integrated diagnostics. As a tool for electrochemical analysis, EIS has now demonstrated real‐time capabilities for tracking shifts in internal resistance, interfacial instability, lithium plating, temperature rise, and gas evolution, which are key precursors to TR. Recent developments in low‐frequency and high‐frequency EIS analysis have enabled the identification of thermal, mechanical, and electrochemical failure modes with increasing resolution.

**FIGURE 11:**
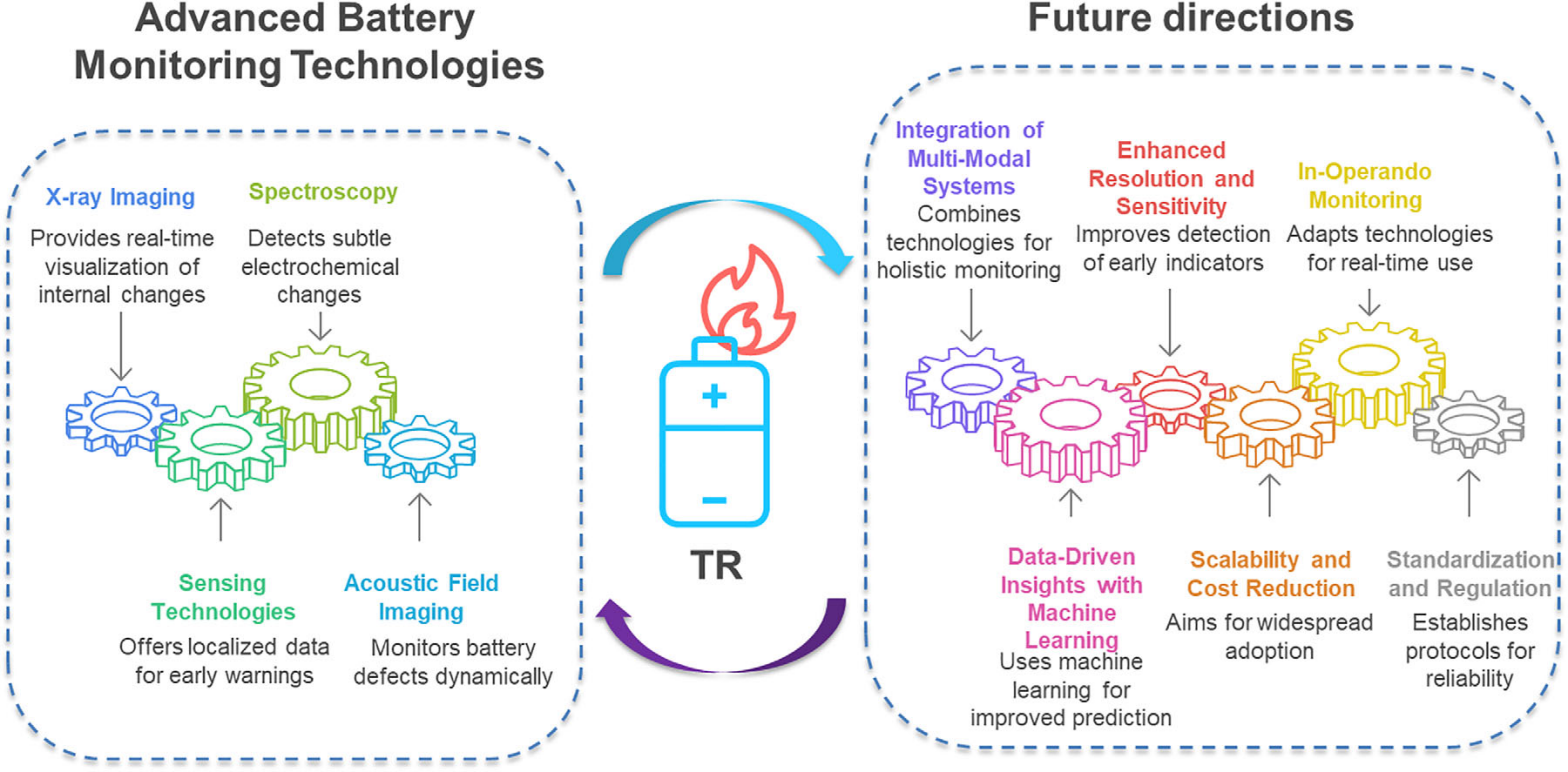
Proposed future directions to monitor battery TR.

For accurate and timely TR prediction, further work correlating across multiple characterization and diagnostic platforms will be highly needed to ensure best practice in battery safety characterization and accelerate its journey to industrial applications, as shown in Figure 11. In detail, developing platforms that combine the strengths of X‐ray imaging, EIS, sensing technologies, and acoustic imaging into cohesive, real‐time diagnostic systems is highly needed. This will enable holistic monitoring and prediction of TR. Then, data‐driven insights from Machine Learning could be used to accurately predict TR, and TR prediction from Machine Learning could be used to accurately predict TR. However, the vast datasets generated by these advanced diagnostics require improved prediction accuracy and failure pattern recognition. In addition, advancing sensor sensitivity and imaging resolution is critical for detecting the earliest indicators of thermal instability under both laboratory and real‐world conditions. Then, to facilitate widespread application, research could aim to simplify and reduce the cost of these diagnostic tools, ensuring they can be seamlessly integrated into commercial battery systems. Last, establishing standardized protocols for the use and interpretation of these diagnostic tools will improve their reliability and facilitate wider industry adoption.

By addressing these challenges, a particularly promising direction for next‐generation battery safety is the development of multi‐modal sensing networks that enable intrinsically ‘smart’ batteries with embedded diagnostic capabilities. In such a framework, fiber‐optic sensors could provide spatially resolved measurements of local temperature and strain, acoustic field imaging could offer non‐invasive tracking of structural heterogeneity, gas evolution, and internal damage, and electrochemical impedance spectroscopy could continuously assess electrochemical state, interfacial stability, and emerging failure precursors. The integration of these complementary data streams would enable a more holistic and reliable evaluation of battery safety status than any single technique alone, thereby supporting a transition from threshold‐based warning to predictive intervention. Realizing this vision, however, will require major advances in sensor integration without compromising cell architecture or ion pathway, robust multimodal data fusion and interpretation, cost‐effective implementation at pack scale, and long‐term operational robustness under realistic cycling and abuse conditions. This integration of advanced diagnostic technologies will deepen our understanding of battery failure modes and ensure safer energy storage solutions, thereby accelerating the global transition toward sustainable energy systems and electric mobility.

## Author Contributions

Yongxiu Chen, Zeyu Sun, Wei Zong, Yuhang Dai, Kai Ling Ng: conceptualization, investigation, writing the original draft, writing the review and editing. Paul Shearing: conceptualization, supervision, writing, review and editing, project administration, and funding acquisition. All authors contributed equally and reviewed, edited, and enriched each section of the paper.

## Conflicts of Interest

The authors declare no conflicts of interest.

## References

[1] M. Meinshausen , J. Lewis , C. McGlade , et al., “Realization of Paris Agreement Pledges May Limit Warming Just Below 2 C,” Nature 604, no. 7905 (2022): 304–309, 10.1038/s41586-022-04553-z. 35418633

[2] L. Peiseler , V. Schenker , K. Schatzmann , S. Pfister , V. Wood , and T. Schmidt , “Carbon Footprint Distributions of Lithium‐Ion Batteries and Their Materials,” Nature Communications 15, no. 1 (2024): 10301, 10.1038/s41467-024-54634-y. PMC11603021 39604365

[3] J. Rogelj , D. Huppmann , V. Krey , et al., “A New Scenario Logic for the Paris Agreement Long‐Term Temperature Goal,” Nature 573, no. 7774 (2019): 357–363, 10.1038/s41586-019-1541-4. 31534246

[4] M. Åhman , Chris Bataille , K. Neuhoff , et al., “A Review of Technology and Policy Deep Decarbonization Pathway Options for Making Energy‐Intensive Industry Production Consistent With the Paris Agreement,” Journal of Cleaner Production 187 (2018): 960–973, 10.1016/j.jclepro.2018.03.107.

[5] C. H. Majesty , “UK Decarbonization Goal Industrial Decarbonisation Strategy. E. I. S. Parliament by the Secretary of State for Business,” in APS *Group on Behalf of the Controller of Her Majesty's Stationery Office* (UK Government, 2021), 6–164.

[6] G. Brunklaus , P. Lennartz , and M. Winter , “Metal Electrodes for Next‐Generation Rechargeable Batteries,” Nature Reviews Electrical Engineering 1, no. 2 (2024): 79–92, 10.1038/s44287-023-00006-5.

[7] M. Fichtner , K. Edström , E. Ayerbe , et al., “Rechargeable Batteries of the Future—The State of the Art From a BATTERY 2030+ Perspective,” Advanced Energy Materials 12, no. 17 (2021): 2102904, 10.1002/aenm.202102904.

[8] S. Flandrois and B. Simon , “Carbon Materials for Lithium‐Ion Rechargeable Batteries,” Carbon 37 (1999): 165–180.

[9] T. M. M. Heenan , A. V. Llewellyn , A. S. Leach , et al., “Resolving Li‐Ion Battery Electrode Particles Using Rapid Lab‐Based X‐Ray Nano‐Computed Tomography for High‐Throughput Quantification,” Advanced Science 7, no. 12 (2020): 2000362, 10.1002/advs.202000362. 32596123 PMC7312274

[10] P. G. Bruce , B. Scrosati , and J. M. Tarascon , “Nanomaterials for Rechargeable Lithium Batteries,” Angewandte Chemie International Edition 47, no. 16 (2008): 2930–2946, 10.1002/anie.200702505. 18338357

[11] F. Cheng , J. Liang , Z. Tao , and J. Chen , “Functional Materials for Rechargeable Batteries,” Advanced Materials 23, no. 15 (2011): 1695–1715, 10.1002/adma.201003587. 21394791

[12] M. Elliott , L. G. Swan , M. Dubarry , and G. Baure , “Degradation of Electric Vehicle Lithium‐Ion Batteries in Electricity Grid Services,” Journal of Energy Storage 32 (2020): 101873, 10.1016/j.est.2020.101873.

[13] H. Xia , Y. Hu , Z. Li , H. Lan , and J. Zhang , “Electron Spin Polarization in Rechargeable Batteries: Theoretical Foundation and Practical Applications,” Advanced Functional Materials 35, no. 3 (2024): 2413491, 10.1002/adfm.202413491.

[14] R. Bubbico , V. Greco , and C. Menale , “Hazardous Scenarios Identification for Li‐Ion Secondary Batteries,” Safety Science 108 (2018): 72–88, 10.1016/j.ssci.2018.04.024.

[15] J. T. Kim , H. Su , Y. Zhong , et al., “All‐Solid‐State Lithium–Sulfur Batteries Through a Reaction Engineering Lens,” Nature Chemical Engineering 1, no. 6 (2024): 400–410, 10.1038/s44286-024-00079-5.

[16] L. Li , J. Yang , R. Tan , et al., “Large‐Scale Current Collectors for Regulating Heat Transfer and Enhancing Battery Safety,” Nature Chemical Engineering 1, no. 8 (2024): 542–551, 10.1038/s44286-024-00103-8.

[17] D. P. Finegan , E. Darcy , M. Keyser , et al., “Identifying the Cause of Rupture of Li‐Ion Batteries During Thermal Runaway,” Advanced Science 5, no. 1 (2018): 1700369, 10.1002/advs.201700369. 29375967 PMC5770664

[18] J. Allen , “Review of Polymers in the Prevention of Thermal Runaway in Lithium‐Ion Batteries,” Energy Reports 6 (2020): 217–224, 10.1016/j.egyr.2020.03.027.

[19] W. L. Kai Liu , Y. Qiu , B. Kong , et al., “Electrospun Core‐Shell Microfiber Separator With Thermal‐Triggered Flame‐Retardant Properties for Lithium‐Ion Batteries,” Science Advances 3 (2017): 1601978. 10.1126/sciadv.1601978 PMC5235334 28097221

[20] Z. Liu , Y. Peng , T. Meng , L. Yu , S. Wang , and X. Hu , “Thermal‐Triggered Fire‐Extinguishing Separators by Phase Change Materials for High‐Safety Lithium‐Ion Batteries,” Energy Storage Materials 47 (2022): 445–452, 10.1016/j.ensm.2022.02.020.

[21] I. T. Song , J. Kang , J. Koh , et al., “Thermal Runaway Prevention Through Scalable Fabrication of Safety Reinforced Layer in Practical Li‐Ion Batteries,” Nature Communications 15, no. 1 (2024): 8294, 10.1038/s41467-024-52766-9. PMC11437208 39333098

[22] R. Gond , W. van Ekeren , R. Mogensen , A. J. Naylor , and R. Younesi , “Non‐Flammable Liquid Electrolytes for Safe Batteries,” Materials Horizons 8, no. 11 (2021): 2913–2928, 10.1039/d1mh00748c. 34549211

[23] J. Hou , L. Wang , X. Feng , et al., “Thermal Runaway of Lithium‐Ion Batteries Employing Flame‐Retardant Fluorinated Electrolytes,” Energy & Environmental Materials 6, no. 1 (2022): 12297, 10.1002/eem2.12297.

[24] J. Hou , L. Lu , L. Wang , et al., “Thermal Runaway of Lithium‐Ion Batteries Employing LiN(SO2F)2‐Based Concentrated Electrolytes,” Nature Communications 11, no. 1 (2020): 5100, 10.1038/s41467-020-18868-w. PMC7547674 33037217

[25] Y. Wu , X. Feng , M. Yang , et al., “Thermal Runaway of Nonflammable Localized High‐Concentration Electrolytes for Practical LiNi_0.8_ Mn_0.1_ Co_0.1_ O_2_ |Graphite‐SiO Pouch Cells,” Advanced Science 9, no. 32 (2022): 2204059, 10.1002/advs.202204059. 36073818 PMC9661853

[26] X. Rui , D. Ren , X. Liu , et al., “Distinct Thermal Runaway Mechanisms of Sulfide‐Based All‐Solid‐State Batteries,” Energy & Environmental Science 16, no. 8 (2023): 3552–3563, 10.1039/d3ee00084b.

[27] M. C. Appleberry , J. A. Kowalski , S. A. Africk , et al., “Avoiding Thermal Runaway in Lithium‐Ion Batteries Using Ultrasound Detection of Early Failure mechanisms,” Journal of Power Sources 535 (2022): 231423, 10.1016/j.jpowsour.2022.231423.

[28] D. P. Finegan , M. Scheel , J. B. Robinson , et al., “In‐Operando High‐Speed Tomography of Lithium‐Ion Batteries During Thermal Runaway,” Nature Communications 6 (2015): 6924, 10.1038/ncomms7924. PMC4423228 25919582

[29] Q. Yu , Y. Yang , A. Tang , et al., “Unsupervised Learning for Lithium‐Ion Batteries Fault Diagnosis and Thermal Runaway Early Warning in Real‐World Electric Vehicles,” Journal of Energy Storage 109 (2025): 115194, 10.1016/j.est.2024.115194.

[30] M. Chen , “Thermal Safety of Lithium‐Ion Batteries: Current Status and Future Trends,” Batteries 11, no. 3 (2025): 112, 10.3390/batteries11030112.

[31] X. Feng , D. Ren , X. He , and M. Ouyang , “Mitigating Thermal Runaway of Lithium‐Ion Batteries,” Joule 4, no. 4 (2020): 743–770, 10.1016/j.joule.2020.02.010.

[32] Y. Wang , X. Feng , W. Huang , X. He , L. Wang , and M. Ouyang , “Challenges and Opportunities to Mitigate the Catastrophic Thermal Runaway of High‐Energy Batteries,” Advanced Energy Materials 13, no. 15 (2023): 2203841, 10.1002/aenm.202203841.

[33] D. Kong , H. Lv , P. Ping , and G. Wang , “A review of Early Warning Methods of Thermal Runaway of Lithium Ion Batteries,” Journal of Energy Storage 64 (2023): 107073, 10.1016/j.est.2023.107073.

[34] S. Dey , Z. A. Biron , S. Tatipamula , et al., “Model‐Based Real‐Time Thermal Fault Diagnosis of Lithium‐Ion Batteries,” Control Engineering Practice 56 (2016): 37–48, 10.1016/j.conengprac.2016.08.002.

[35] L. Jiang , Z. Deng , X. Tang , L. Hu , X. Lin , and X. Hu , “Data‐Driven Fault Diagnosis and Thermal Runaway Warning for Battery Packs Using Real‐World Vehicle Data,” Energy 234 (2021): 121266, 10.1016/j.energy.2021.121266.

[36] IEA, IEA, Paris Licence: CC BY 4.0 , 2024, https://www.iea.org/reports/global‐ev‐outlook‐2024.

[37] D. Ren , X. Feng , L. Liu , et al., “Investigating the Relationship Between Internal Short Circuit and Thermal Runaway of Lithium‐Ion Batteries Under Thermal Abuse Condition,” Energy Storage Materials 34 (2021): 563–573, 10.1016/j.ensm.2020.10.020.

[38] X. Feng , M. Ouyang , X. Liu , L. Lu , Y. Xia , and X. He , “Thermal Runaway Mechanism of Lithium Ion Battery for Electric Vehicles: A Review,” Energy Storage Materials 10 (2018): 246–267, 10.1016/j.ensm.2017.05.013.

[39] C. F. Lopez , J. A. Jeevarajan , and P. P. Mukherjee , “Experimental Analysis of Thermal Runaway and Propagation in Lithium‐Ion Battery Modules,” Journal of the Electrochemical Society 162, no. 9 (2015): A1905, 10.1149/2.0921509jes.

[40] L. Liu , X. Feng , M. Zhang , et al., “Comparative Study on Substitute Triggering Approaches for Internal Short Circuit in Lithium‐Ion Batteries,” Applied Energy 259 (2020): 114143, 10.1016/j.apenergy.2019.114143.

[41] Z. Chai , J. Li , Z. Liu , Z. Liu , and X. Jin , “Experimental Analysis and Safety Assessment of Thermal Runaway Behavior in Lithium Iron Phosphate Batteries Under Mechanical Abuse,” Scientific Reports 14, no. 1 (2024): 8673, 10.1038/s41598-024-58891-1. 38622171 PMC11018818

[42] H. Wang , Z. Yang , C. Jiang , Z. Ji , and Z. Zhu , “Internal Temperature and Flame Jet Characteristics During Thermal Runaway Triggered by Nail Penetration for NCM811 Lithium‐Ion Battery,” Journal of Thermal Analysis and Calorimetry 147, no. 24 (2022): 14925–14938, 10.1007/s10973-022-11677-x.

[43] J. Zhu , X. Zhang , E. Sahraei , and T. Wierzbicki , “Deformation and Failure Mechanisms of 18650 Battery Cells Under Axial Compression,” Journal of Power Sources 336 (2016): 332–340, 10.1016/j.jpowsour.2016.10.064.

[44] H. Maleki and J. N. Howard , “Internal Short Circuit in Li‐Ion Cells,” Journal of Power Sources 191, no. 2 (2009): 568–574, 10.1016/j.jpowsour.2009.02.070.

[45] D. P. Finegan , J. Billman , J. Darst , et al., “The Battery Failure Databank: Insights From an Open‐Access Database of Thermal Runaway Behaviors of Li‐Ion Cells and a Resource for Benchmarking Risks,” Journal of Power Sources 597 (2024): 234106, 10.1016/j.jpowsour.2024.234106.

[46] W. Q. Walker , K. Cooper , P. Hughes , et al., “The Effect of Cell Geometry and Trigger Method on the Risks Associated With Thermal Runaway of Lithium‐Ion Batteries,” Journal of Power Sources 524 (2022): 230645, 10.1016/j.jpowsour.2021.230645.

[47] National Standard of the People's Republic of China , Electric Vehicles Traction Battery Safety Requirements, (State Administration for Market Regulation, National Standardization Administration, 2020): 57.

[48] Candidate Alternative Test Methods for the Internal Short Circuit Test of IEC 62660‐3 (IEC, 2017), 1–12.

[49] T. D. Hatchard , S. Trussler , and J. R. Dahn , “Building a “Smart Nail” for Penetration Tests on Li‐Ion Cells,” Journal of Power Sources 247 (2014): 821–823, 10.1016/j.jpowsour.2013.09.022.

[50] D. P. Finegan , B. Tjaden , T. M. M. Heenan , et al., “Tracking Internal Temperature and Structural Dynamics During Nail Penetration of Lithium‐Ion Cells,” Journal of the Electrochemical Society 164, no. 13 (2017): A3285–A3291, 10.1149/2.1501713jes.

[51] J. Diekmann , S. Doose , S. Weber , S. Münch , W. Haselrieder , and A. Kwade , “Development of a New Procedure for Nail Penetration of Lithium‐Ion Cells to Obtain Meaningful and Reproducible Results,” Journal of the Electrochemical Society 167, no. 9 (2020): 090504, 10.1149/1945-7111/ab78ff.

[52] S. Huang , Z. Du , Q. Zhou , K. Snyder , S. Liu , and G. Zhang , “In Situ Measurement of Temperature Distributions in a Li‐Ion Cell During Internal Short Circuit and Thermal Runaway,” Journal of the Electrochemical Society 168, no. 9 (2021): 090510, 10.1149/1945-7111/ac1d7b.

[53] Z. Sun , E. Read , Y. Chen , Y. Dai , J. Marco , and P. R. Shearing , “Numerical and Experimental Characterization of Nail Penetration Induced Thermal Runaway Propagation in 21 700 Lithium‐Ion Batteries: Exploring the Role of Interstitial Thermal Barrier Materials,” Journal of Energy Chemistry 109 (2025): 576–589, 10.1016/j.jechem.2025.05.037.

[54] L. Zhang , Y. Liu , X. Huang , and X. Huang , “Intra‐cell thermal runaway propagation Within a Cylindrical Battery Induced by Nail Penetration,” International Journal of Thermal Sciences 210 (2025): 109633, 10.1016/j.ijthermalsci.2024.109633.

[55] M. Yang , M. Rong , Y. Ye , et al., “A Comprehensive Study of Thermal Runaway Behavior and Early Warning Subjected to Internal Short‐Circuit,” Journal of Power Sources 620 (2024): 235213, 10.1016/j.jpowsour.2024.235213.

[56] H. Chen , E. Kalamaras , A. Abaza , Y. Tripathy , J. Page , and A. Barai , “Experimental Study of Sidewall Rupture of Cylindrical Lithium‐Ion Batteries Under Radial Nail Penetration,” Journal of the Electrochemical Society 169, no. 12 (2022): 120528, 10.1149/1945-7111/acadac.

[57] G. Zhang , X. Wei , X. Tang , J. Zhu , S. Chen , and H. Dai , “Internal Short Circuit Mechanisms, Experimental Approaches and Detection Methods of Lithium‐Ion Batteries for Electric Vehicles: A Review,” Renewable and Sustainable Energy Reviews 141 (2021): 110790, 10.1016/j.rser.2021.110790.

[58] C. Zhan , J. Lu , A. Jeremy Kropf , et al., “Mn(II) Deposition on Anodes and its Effects on Capacity Fade in Spinel Lithium Manganate–Carbon Systems,” Nature Communications 4 (2013): 2437, 10.1038/ncomms3437. 24077265

[59] H. Shin , J. Park , A. M. Sastry , and W. Lu , “Degradation of the Solid Electrolyte Interphase Induced by the Deposition of Manganese Ions,” Journal of Power Sources 284 (2015): 416–427, 10.1016/j.jpowsour.2015.03.039.

[60] J. Liu , Z. Wang , J. Bai , T. Gao , and N. Mao , “Heat Generation and Thermal Runaway Mechanisms Induced by Overcharging of Aged Lithium‐Ion Battery,” Applied Thermal Engineering 212 (2022): 118565, 10.1016/j.applthermaleng.2022.118565.

[61] K. Wang , D. Wu , C. Chang , J. Zhang , D. Ouyang , and X. Qian , “Charging Rate Effect on Overcharge‐Induced Thermal Runaway Characteristics and Gas Venting Behaviors for Commercial Lithium Iron Phosphate Batteries,” Journal of Cleaner Production 434 (2024): 139992, 10.1016/j.jclepro.2023.139992.

[62] S. Sharifi‐Asl , J. Lu , K. Amine , and R. Shahbazian‐Yassar , “Oxygen Release Degradation in Li‐Ion Battery Cathode Materials: Mechanisms and Mitigating Approaches,” Advanced Energy Materials 9, no. 22 (2019): 1900551, 10.1002/aenm.201900551.

[63] H.‐Z. Jin , X.‐F. Han , P. M. Radjenovic , J.‐H. Tian , and J.‐F. Li , “Facile and Effective Positive Temperature Coefficient (PTC) Layer for Safer Lithium‐Ion Batteries,” Journal of Physical Chemistry C 125, no. 3 (2021): 1761–1766, 10.1021/acs.jpcc.0c10196.

[64] Y. Liu , L. Qin , Y. Wu , X. Wu , and W. Jin , “Thermal Runaway‐Induced Current Interrupt Device and Vent Activation Behaviour in an 18650 Lithium‐Ion Battery Cap Using the Johnson‐Cook Criterion,” Journal of Energy Storage 68 (2023): 107879, 10.1016/j.est.2023.107879.

[65] S. Tang , Y. Liang , C. Zhong , et al., “Revisiting the Overdischarge Process as a Novel Accelerated Aging Method for LiFePO_4_/Graphite Batteries Through the Unveiling of SEI Evolution Mechanism,” Energy Storage Materials 74 (2025): 103916, 10.1016/j.ensm.2024.103916.

[66] Z. Guo , S. Yang , W. Zhao , et al., “Overdischarge‐Induced Evolution of Cu Dendrites and Degradation of Mechanical Properties in Lithium‐Ion Batteries,” Journal of Energy Chemistry 78 (2023): 497–506, 10.1016/j.jechem.2022.12.013.

[67] R. Guo , L. Lu , M. Ouyang , and X. Feng , “Mechanism of the Entire Overdischarge Process and Overdischarge‐Induced Internal Short Circuit in Lithium‐Ion Batteries,” Scientific Reports 6 (2016): 30248, 10.1038/srep30248. 27444934 PMC4957210

[68] T. Langner , T. Sieber , and J. Acker , “Studies on the Deposition of Copper in Lithium‐Ion Batteries During the Deep Discharge Process,” Scientific Reports 11, no. 1 (2021): 6316, 10.1038/s41598-021-85575-x. 33737549 PMC7973563

[69] D. Ouyang , J. Weng , M. Chen , J. Wang , and Z. Wang , “Sensitivities of Lithium‐Ion Batteries With Different Capacities to Overcharge/Over‐Discharge,” Journal of Energy Storage 52 (2022): 104997, 10.1016/j.est.2022.104997.

[70] S. Hildebrand , A. Eddarir , and N. Lebedeva , Overview of Battery Safety Tests in Standards for Stationary Battery Energy Storage Systems (Publications Office of the European Union, 2024).

[71] Z. An , T. Shi , Y. Zhao , et al., “Study on Aging and External Short Circuit Mechanisms of Li‐Ion Cells With Different Electrode Thicknesses,” Applied Energy 350 (2023): 121796, 10.1016/j.apenergy.2023.121796.

[72] Y. Jia and J. Xu , “Data‐Driven Short Circuit Resistance Estimation in Battery Safety Issues,” Journal of Energy Chemistry 79 (2023): 37–44, 10.1016/j.jechem.2022.12.035.

[73] Y. Zhang , P. Ping , X. Dai , et al., “Failure Mechanism and Thermal Runaway Behavior of Lithium‐Ion Battery Induced by Arc Faults,” Renewable Sustainable Energy Reviews 207 (2025): 114914, 10.1016/j.rser.2024.114914.

[74] S. Ko , H. Otsuka , S. Kimura , et al., “Rapid Safety Screening Realized by Accelerating Rate Calorimetry With Lab‐Scale Small Batteries,” Nature Energy 10 (2025): 707–714, 10.1038/s41560-025-01751-7.

[75] X. Feng , M. Fang , X. He , et al., “Thermal Runaway Features of Large Format Prismatic Lithium Ion Battery Using Extended Volume Accelerating Rate Calorimetry,” Journal of Power Sources 255 (2014): 294–301, 10.1016/j.jpowsour.2014.01.005.

[76] M. Buckwell , C. Kirchner‐Burles , R. E. Owen , et al., “Failure and Hazard Characterisation of High‐Power Lithium‐Ion Cells via Coupling Accelerating Rate Calorimetry With in‐Line Mass Spectrometry, Statistical and Post‐Mortem Analyses,” Journal of Energy Storage 65 (2023): 107069, 10.1016/j.est.2023.107069.

[77] X. Liu , Z. Wu , S. I. Stoliarov , M. Denlinger , A. Masias , and K. Snyder , “Heat Release During Thermally‐Induced Failure of a Lithium Ion Battery: Impact of Cathode Composition,” Fire Safety Journal 85 (2016): 10–22, 10.1016/j.firesaf.2016.08.001.

[78] C. M. R. Vendra , A. V. Shelke , J. E. H. Buston , et al., “Numerical and Experimental Characterisation of High Energy Density 21700 Lithium‐Ion Battery Fires,” Process Safety and Environmental Protection 160 (2022): 153–165, 10.1016/j.psep.2022.02.014.

[79] J. Lamb , L. Torres‐Castro , J. C. Hewson , R. C. Shurtz , and Y. Preger , “Investigating the Role of Energy Density in Thermal Runaway of Lithium‐Ion Batteries With Accelerating Rate Calorimetry,” Journal of the Electrochemical Society 168, no. 6 (2021): 060516, 10.1149/1945-7111/ac0699.

[80] X. Huang , M. Xiao , D. Han , J. Xue , S. Wang , and Y. Meng , “Thermal Runaway Features of Lithium Sulfur Pouch Cells at Various States of Charge Evaluated by Extended Volume‐Accelerating Rate Calorimetry,” Journal of Power Sources 489 (2021): 229503, 10.1016/j.jpowsour.2021.229503.

[81] A. Teng , Y. Li , Y. Zhang , et al., “A New Method to Accurately Measure Lithium‐Ion Battery Specific Heat Capacity With ARC Heating‐Waiting Process,” Fire Technology 61 (2024): 4001–4020, 10.1007/s10694-024-01649-y.

[82] B. Gulsoy , T. A. Vincent , J. E. H. Sansom , and J. Marco , “In‐Situ Temperature Monitoring of a Lithium‐Ion Battery Using an Embedded Thermocouple for Smart Battery Applications,” Journal of Energy Storage 54 (2022): 105260, 10.1016/j.est.2022.105260.

[83] A. Jinasena , L. Spitthoff , M. S. Wahl , et al., “Online Internal Temperature Sensors in Lithium‐Ion Batteries: State‐of‐the‐Art and Future Trends,” Frontiers in Chemical Engineering 4 (2022), 10.3389/fceng.2022.804704.

[84] W. Mei , Z. Liu , C. Wang , et al., “Operando Monitoring of Thermal Runaway in Commercial Lithium‐Ion Cells via Advanced Lab‐on‐Fiber Technologies,” Nature Communications 14, no. 1 (2023): 5251, 10.1038/s41467-023-40995-3. PMC10462619 37640698

[85] X. Kong , L. Lu , Y. Yuan , et al., “Foreign Matter Defect Battery and Sudden Spontaneous Combustion,” eTransportation 12 (2022): 100170, 10.1016/j.etran.2022.100170.

[86] L. David , R. E. Ruther , D. Mohanty , et al., “Identifying Degradation Mechanisms in Lithium‐Ion Batteries With Coating Defects at the Cathode,” Applied Energy 231 (2018): 446–455, 10.1016/j.apenergy.2018.09.073.

[87] Y. Wu , S. Saxena , Y. Xing , et al., “Analysis of Manufacturing‐Induced Defects and Structural Deformations in Lithium‐Ion Batteries Using Computed Tomography,” Energies 11, no. 4 (2018): 925, 10.3390/en11040925.

[88] O. Badmos , A. Kopp , T. Bernthaler , and G. Schneider , “Image‐Based Defect Detection in Lithium‐Ion Battery Electrode Using Convolutional Neural Networks,” Journal of Intelligent Manufacturing 31, no. 4 (2019): 885–897, 10.1007/s10845-019-01484-x.

[89] P. J. Withers , C. Bouman , S. Carmignato , et al., “X‐Ray Computed Tomography,” Nature Reviews Methods Primers 1, no. 1 (2021): 18, 10.1038/s43586-021-00015-4.

[90] D. P. Finegan , M. Scheel , J. B. Robinson , et al., “Investigating Lithium‐Ion Battery Materials During Overcharge‐Induced Thermal Runaway: An Operando and Multi‐Scale X‐Ray CT Study,” Physical Chemistry Chemical Physics 18, no. 45 (2016): 30912–30919, 10.1039/c6cp04251a. 27388638

[91] T. M. M. Heenan , I. Mombrini , A. Llewellyn , et al., “Mapping Internal Temperatures During High‐Rate Battery Applications,” Nature 617, no. 7961 (2023): 507–512, 10.1038/s41586-023-05913-z. 37198308

[92] D. Patel , J. B. Robinson , S. Ball , D. J. L. Brett , and P. R. Shearing , “Thermal Runaway of a Li‐Ion Battery Studied by Combined ARC and Multi‐Length Scale X‐ray CT,” Journal of the Electrochemical Society 167, no. 9 (2020): 090511, 10.1149/1945-7111/ab7fb6.

[93] D. P. Finegan , E. Darcy , M. Keyser , et al., “Characterising Thermal Runaway Within Lithium‐Ion Cells by Inducing and Monitoring Internal Short Circuits,” Energy & Environmental Science 10, no. 6 (2017): 1377–1388, 10.1039/c7ee00385d.

[94] M. T. M. Pham , J. J. Darst , W. Q. Walker , et al., “Prevention of Lithium‐Ion Battery Thermal Runaway Using Polymer‐Substrate Current Collectors,” Cell Reports Physical Science 2, no. 3 (2021): 100360, 10.1016/j.xcrp.2021.100360.

[95] M. Fransson , L. Broche , M. Buckwell , et al., “Sidewall Breach During Lithium‐Ion Battery Thermal Runaway Triggered by Cell‐to‐Cell Propagation Visualized Using High‐Speed X‐Ray Imaging,” Journal of Energy Storage 71 (2023): 108088, 10.1016/j.est.2023.108088.

[96] A. N. P. Radhakrishnan , M. Buckwell , M. Pham , et al., “Quantitative Spatiotemporal Mapping of Thermal Runaway Propagation Rates in Lithium‐Ion Cells Using Cross‐Correlated Gabor Filtering,” Energy & Environmental Science 15, no. 8 (2022): 3503–3518, 10.1039/d1ee03430h.

[97] M. Fransson , J. Pfaff , L. Broche , et al., “Exploring Thermal Runaway Propagation in Li‐Ion Batteries Through High‐Speed X‐Ray Imaging and Thermal Analysis: Impact of Cell Chemistry and Electrical Connections,” Journal of Power Sources 617 (2024): 234916, 10.1016/j.jpowsour.2024.234916.

[98] J. Scharf , M. Chouchane , D. P. Finegan , et al., “Bridging Nano‐ and Microscale X‐Ray Tomography for Battery Research by Leveraging Artificial Intelligence,” Nature Nanotechnology 17, no. 5 (2022): 446–459, 10.1038/s41565-022-01081-9. 35414116

[99] S. Hao , S. R. Daemi , T. M. M. Heenan , et al., “Fast Degradation of Solid Electrolyte in Initial Cycling Processes, Tracked in 3D by Synchrotron X‐Ray Computed Tomography,” ACS Nano 19, no. 22 (2025): 20516–20525, 10.1021/acsnano.4c17739. 40432194 PMC12164515

[100] S. Hao , Q. Zhang , X. Kong , Z. Wang , X. P. Gao , and P. R. Shearing , “Intrinsic Mechanical Parameters and Their Characterization in Solid‐State Lithium Batteries,” Advanced Energy Materials 15, no. 11 (2024): 2404384, 10.1002/aenm.202404384.

[101] R. Lin , S. M. Bak , Y. Shin , et al., “Hierarchical Nickel Valence Gradient Stabilizes High‐Nickel Content Layered Cathode Materials,” Nature Communications 12, no. 1 (2021): 2350, 10.1038/s41467-021-22635-w. PMC8058063 33879789

[102] H. Michael , F. Iacoviello , T. Heenan , et al., “A Dilatometric Study of Graphite Electrodes During Cycling With X‐Ray Computed Tomography,” Journal of the Electrochemical Society 168, no. 1 (2021): 010507, 10.1149/1945-7111/abd648.

[103] H. C. W. Parks , M. P. Jones , A. Wade , et al., “Non‐Linear Cracking Response to Voltage Revealed by Operando X‐Ray Tomography in Polycrystalline NMC811†,” EES Batteries 1, no. 3 (2025): 482–494, 10.1039/d5eb00008d.

[104] Y. Peng , L. Zhou , M. Van Winkle , et al., “Operando X‐ray imaging reveals size‐dependent Evolution of Cobalt Oxide Thermochemical Material During Thermal Redox Cycles,” Nature Communications 16 (2025): 11278. 10.1038/s41467-025-66174-0 PMC12717218 41407672

[105] V. Vanpeene , O. Stamati , C. Guilloud , et al., “Comparative Study of the Quantitative Analysis of Battery Materials With X‐Ray Nano‐Tomography: From Ex Situ Toward Operando Measurements,” ACS Nano 19, no. 10 (2025): 9994–10012, 10.1021/acsnano.4c16419. 40040237

[106] S. Hao , Q. Zhang , X. Kong , Z. Wang , X.‐P. Gao , and P. R. Shearing , “Intrinsic Mechanical Parameters and Their Characterization in Solid‐State Lithium Batteries,” Advanced Energy Materials 11 (2025): 2404384, 10.1002/aenm.202404384.

[107] E. Allen , L. Y. Lim , X. Xiao , et al., “Spatial Quantification of Microstructural Degradation During Fast Charge in 18650 Lithium‐Ion Batteries Through Operando X‐Ray Microtomography and Euclidean Distance Mapping,” ACS Applied Energy Materials 5, no. 10 (2022): 12798–12808, 10.1021/acsaem.2c02397.

[108] M. H. Parekh , B. Li , M. Palanisamy , T. E. Adams , V. Tomar , and V. G. Pol , “In Situ Thermal Runaway Detection in Lithium‐Ion Batteries With an Integrated Internal Sensor,” ACS Applied Energy Materials 3, no. 8 (2020): 7997–8008, 10.1021/acsaem.0c01392.

[109] M. Nascimento , S. Novais , M. S. Ding , et al., “Internal Strain and Temperature Discrimination With Optical Fiber Hybrid Sensors in Li‐Ion Batteries,” Journal of Power Sources 410‐411 (2019): 1–9, 10.1016/j.jpowsour.2018.10.096.

[110] Y. Shen , S. Wang , H. Li , K. Wang , and K. Jiang , “An Overview on In Situ/Operando Battery Sensing Methodology Through Thermal and Stress Measurements,” Journal of Energy Storage 64 (2023): 107164, 10.1016/j.est.2023.107164.

[111] W. Wang , Y. Zhang , B. Xie , et al., “Deciphering Advanced Sensors for Life and Safety Monitoring of Lithium Batteries,” Advanced Energy Materials 14, no. 24 (2024): 2304173, 10.1002/aenm.202304173.

[112] S. Koch , K. Birke , and R. Kuhn , “Fast Thermal Runaway Detection for Lithium‐Ion Cells in Large Scale Traction Batteries,” Batteries 4, no. 2 (2018): 16, 10.3390/batteries4020016.

[113] J. Bang , B. Chun , M. Kim , J. Lim , Y. Han , and H. So , “Rapid Thermal Runaway Detection of Lithium‐Ion Battery via Swelling‐Based State‐of‐Charge Monitoring Using Piezoresistive Sponge Sensor,” eTransportation 24 (2025): 100404, 10.1016/j.etran.2025.100404.

[114] L. Wang , W. Choi , K. Yoo , K. Nam , T. J. Ko , and J. Choi , “Stretchable Carbon Nanotube Dilatometer for In Situ Swelling Detection of Lithium‐Ion Batteries,” ACS Applied Energy Materials 3, no. 4 (2020): 3637–3644, 10.1021/acsaem.0c00114.

[115] T. Cai , S. Pannala , A. G. Stefanopoulou , and J. B. Siegel , “Battery Internal Short Detection Methodology Using Cell Swelling Measurements,” *American Control Conference (ACC), Denver, CO, USA* (2020): pp. 1143–1148, https://doi/org/10.23919/ACC45564.2020.9147956.

[116] X. Chen , L. Gan , and X. Guo , “Optical Fiber‐Based Gas Sensing for Early Warning of Thermal Runaway in Lithium‐Ion Batteries,” Advanced Sensor Research 2, no. 12 (2023): 2300055, 10.1002/adsr.202300055.

[117] Y. Li , L. Wang , Y. Song , W. Wang , C. Lin , and X. He , “Functional Optical Fiber Sensors Detecting Imperceptible Physical/Chemical Changes for Smart Batteries,” Nanomicro Letters 16, no. 1 (2024): 154, 10.1007/s40820-024-01374-9. PMC10948733 38499708

[118] Y. Jin , Z. Zheng , D. Wei , et al., “Detection of Micro‐Scale Li Dendrite via H_2_ Gas Capture for Early Safety Warning,” Joule 4, no. 8 (2020): 1714–1729, 10.1016/j.joule.2020.05.016.

[119] T. Cai , A. G. Stefanopoulou , and J. B. Siegel , “Early Detection for Li‐Ion Batteries Thermal Runaway Based on Gas Sensing,” ECS Transactions 89 (2019): 85–97, 10.1149/08901.0085ecst.

[120] B. Gulsoy , H. Chen , C. Briggs , T. A. Vincent , J. E. H. Sansom , and J. Marco , “Real‐Time Simultaneous Monitoring of Internal Temperature and Gas Pressure in Cylindrical Cells During Thermal Runaway,” Journal of Power Sources 617 (2024): 235147, 10.1016/j.jpowsour.2024.235147.

[121] B. Gulsoy , T. A. Vincent , C. Briggs , J. E. H. Sansom , and J. Marco , “In‐Situ Measurement of Internal Gas Pressure Within Cylindrical Lithium‐Ion Cells,” Journal of Power Sources 570 (2023): 233064, 10.1016/j.jpowsour.2023.233064.

[122] A. G. Hsieh , S. Bhadra , B. J. Hertzberg , et al., “Electrochemical‐Acoustic Time of flight: In Operando Correlation of Physical Dynamics With Battery Charge and Health,” Energy & Environmental Science 8, no. 5 (2015): 1569–1577, 10.1039/c5ee00111k.

[123] J. B. Robinson , M. Maier , G. Alster , T. Compton , D. J. L. Brett , and P. R. Shearing , “Spatially Resolved Ultrasound Diagnostics of Li‐Ion Battery Electrodes,” Physical Chemistry Chemical Physics 21, no. 12 (2019): 6354–6361, 10.1039/c8cp07098a. 30601492

[124] H. Huo , K. Huang , W. Luo , et al., “Evaluating Interfacial Stability in Solid‐State Pouch Cells via Ultrasonic Imaging,” ACS Energy Letters 7, no. 2 (2022): 650–658, 10.1021/acsenergylett.1c02363.

[125] Q. Ke , S. Jiang , W. Li , W. Lin , X. Li , and H. Huang , “Potential of Ultrasonic Time‐of‐Flight and Amplitude as the Measurement for State of Charge and Physical Changings of Lithium‐Ion Batteries,” Journal of Power Sources 549 (2022): 232031, 10.1016/j.jpowsour.2022.232031.

[126] H. Popp , M. Koller , S. Keller , G. Glanz , R. Klambauer , and A. Bergmann , “State Estimation Approach of Lithium‐Ion Batteries by Simplified Ultrasonic Time‐of‐Flight Measurement,” IEEE Access 7 (2019): 170992–171000, 10.1109/access.2019.2955556.

[127] T. M. McGee , B. Neath , S. Matthews , O. A. Ezekoye , and M. R. Haberman , “Ultrasonic Inspection of Lithium‐Ion Pouch Cells Subjected to Localized Thermal Abuse,” Journal of Power Sources 583 (2023): 233542, 10.1016/j.jpowsour.2023.233542.

[128] A. Fordham , Z. Milojevic , E. Giles , et al., “Correlative Non‐Destructive Techniques to Investigate Aging and Orientation Effects in Automotive Li‐Ion Pouch Cells,” Joule 7, no. 11 (2023): 2622–2652, 10.1016/j.joule.2023.10.011.

[129] R. J. Copley , D. Cumming , Y. Wu , and R. S. Dwyer‐Joyce , “Measurements and Modelling of the Response of an Ultrasonic Pulse to a Lithium‐Ion Battery as a Precursor for State of Charge Estimation,” Journal of Energy Storage 36 (2021): 102406, 10.1016/j.est.2021.102406.

[130] P.‐S. Ma , H. Lee , and Y.‐H. Seo , “Identifying Ultrasonic Scattering From Multi‐Layered Lithium‐Ion Battery Cells: Mechanical Modeling and Experimental Validation,” Journal of Energy Storage 92 (2024): 112077, 10.1016/j.est.2024.112077.

[131] Y. Ren , M. Huang , G. Liu , et al., “Decoding Coupled Mechanical–Electrochemical Responses in Multi‐Layer Batteries via Generalized Ultrasonic Dynamics,” Energy Storage Materials 82 (2025): 104618, 10.1016/j.ensm.2025.104618.

[132] Y. Pan , K. Xu , R. Wang , H. Wang , G. Chen , and K. Wang , “Lithium‐Ion Battery Condition Monitoring: A Frontier in Acoustic Sensing Technology,” Energies 18, no. 5 (2025): 1068, 10.3390/en18051068.

[133] E. Galiounas , T. G. Tranter , R. E. Owen , J. B. Robinson , P. R. Shearing , and D. J. L. Brett , “Battery State‐of‐Charge Estimation Using Machine Learning Analysis of Ultrasonic Signatures,” Energy and AI 10 (2022): 100188, 10.1016/j.egyai.2022.100188.

[134] P. Dong , Z. Liu , P. Wu , Z. Li , Z. Wang , and J. Zhang , “Reliable and Early Warning of Lithium‐Ion Battery Thermal Runaway Based on Electrochemical Impedance Spectrum,” Journal of the Electrochemical Society 168, no. 9 (2021): 090529, 10.1149/1945-7111/ac239b.

[135] Y. Li , L. Jiang , N. Zhang , et al., “Early Warning Method for Thermal Runaway of Lithium‐Ion Batteries Under Thermal Abuse Condition Based on Online Electrochemical Impedance Monitoring,” Journal of Energy Chemistry 92 (2024): 74–86, 10.1016/j.jechem.2023.12.049.

[136] J. G. Zhu , Z. C. Sun , X. Z. Wei , and H. F. Dai , “A New Lithium‐Ion Battery Internal Temperature on‐Line Estimate Method Based on Electrochemical Impedance Spectroscopy Measurement,” Journal of Power Sources 274 (2015): 990–1004, 10.1016/j.jpowsour.2014.10.182.

[137] N. S. Spinner , C. T. Love , S. L. Rose‐Pehrsson , and S. G. Tuttle , “Expanding the Operational Limits of the Single‐Point Impedance Diagnostic for Internal Temperature Monitoring of Lithium‐Ion Batteries,” Electrochimica Acta 174 (2015): 488–493, 10.1016/j.electacta.2015.06.003.

